# Fabrication of Stable Liquid-like Wetting Buckled Surfaces as Bioinspired Antibiofouling Coatings by Using Silicon-Containing Block Copolymers

**DOI:** 10.1021/acsami.4c06172

**Published:** 2024-07-04

**Authors:** Ting-Lun Chen, Ching-Yu Huang, Yi-Shan Lai, Yi-Chen Chen, Yi-Ju Yang, Wei-Lung Wang, Han-Yu Hsueh

**Affiliations:** † Department of Materials Science and Engineering, National Chung Hsing University, Taichung, Taiwan 40227, Republic of China; ‡ Innovation and Development Center of Sustainable Agriculture, National Chung Hsing University, Taichung, Taiwan 40227, Republic of China; § Department of Natural Resources and Environmental Studies, National Dong Hwa University, Hualien, Taiwan 974301, Republic of China; ∥ Department of Biology, National Changhua University of Education, Changhua, Taiwan 50007, Republic of China

**Keywords:** slippery, antibiofouling, block copolymer, buckle, sustainability

## Abstract

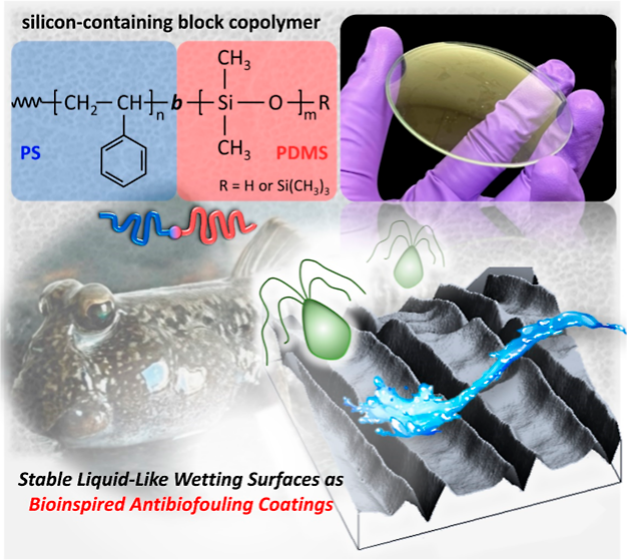

Inspired by animals with a slippery epidermis, durable slippery antibiofouling coatings with liquid-like wetting buckled surfaces are successfully constructed in this study by combining dynamic-interfacial-release-induced buckling with self-assembled silicon-containing diblock copolymer (diBCP). The core diBCP material is polystyrene-*block*-poly(dimethylsiloxane) (PS-*b*-PDMS). Because silicon-containing polymers with intrinsic characters of low surface energy, they easily flow over and cover a surface after it has undergone controlled thermal treatment, generating a slippery wetting layer on which can eliminate polar interactions with biomolecules. Additionally, microbuckled patterns result in curved surfaces, which offer fewer points at which organisms can attach to the surface. Different from traditional slippery liquid-infused porous surfaces, the proposed liquid-like PDMS wetting layer, chemically bonded with PS, is stable and slippery but does not flow away. PS-*b*-PDMS diBCPs with various PDMS volume fractions are studied to compare the influence of PDMS segment length on antibiofouling performance. The surface characteristics of the diBCPs—ease of processing, transparency, and antibiofouling, anti-icing, and self-cleaning abilities—are examined under various conditions. Being able to fabricate ecofriendly silicon-based lubricant layers without needing to use fluorinated compounds and costly material precursors is an advantage in industrial practice.

## Introduction

Marine biofouling refers to the detrimental accumulation and growth of biomolecules and organisms on various marine surfaces that causes damage to the structure and function of those surfaces. Marine biofouling is a major issue that affects a range of things, from ship maintenance to ecosystem safety, including in freshwater and other marine environments. Biofouling roughens the hulls of ships, increasing their drag. This results in greater fuel consumption and also causes issues with instrument functioning of ship instruments and the maneuverability of ships.^1,2^ In 2011, typical levels of biofouling cost the US Navy an estimated US$56 million a year in additional fuel and maintenance costs for all ships of the Arleigh Burke (DDG-51) class. The cost could increase to a total of US$119 million for heavier fouling.^3^ According to the Clean Shipping Coalition, poor hull and propeller performance accounts for approximately 10% of the world fleet’s energy consumption, and fouled hulls cost the global shipping industry as much as US$30 billion in additional fuel costs every year.^4^ The use of fuel attributed to biofouling was predicted to increase CO_2_ and SO_2_ emissions by between 38 and 72% by 2020. In addition to the cost dimension of the biofouling problem, a coating of barnacles, algae, and slimy gunk on ships can serve as the mechanism underlying the introduction of invasive species, which can greatly damage local ecosystems.^5^ Therefore, approaches for effectively inhibiting biofouling are urgently required.

The development of antibiofouling coatings has attracted considerable research attention, and various antibiofouling coatings have been developed. The use of antibiofouling coatings is the most effective, economical, and widely used antibiofouling strategy.^6^ Hydrophilic polymers such as poly(ethylene glycol) are the most extensively used materials because their surface energy is similar to that of water (72 mN m^–1^).^7−10^ Using a material with this property minimizes the interfacial energy between the surface and water, meaning that from the energetic perspective, the material surface prefers to be in contact with water than with amphiphilic biomolecules, such as proteins. Hydrophilic polymers can order water molecules and stabilize them such that they form a thin water film on the polymer surface, enhancing the resistance of water to displacement. However, hydrophilic polymers lack long-term stability because they oxidize and microbially degrade, and increasing their stability is difficult.^11−13^ Recent research has focused on charged structures. Zwitterions and polyelectrolytes are popular candidates for charged structures because they allow for tightly bound and highly structured water layers to form on them.^14−18^ Although hydrophilic coatings have shown immense promise as antibiofouling agents, once organisms have adhered to them, they cannot easily be removed and they render the hydrophilic coatings nonfunctional.

Low-surface-energy materials [e.g., polydimethylsiloxane (PDMS) and fluorinated molecules] with specific geometric topographies are antibiofouling coatings that are different from those in which water layers form; these are considered to be the next potentially ideal candidates for biofouling release coatings.^19−23^ Aizenberg et al. pioneered a new type of antibiofouling surface [i.e., slippery liquid-infused porous surface (SLIPS)] by infusing a rough and porous polytetrafluoroethylene substrate with an oil-based lubricant to mimic *Nepenthes* plants.^24^ In our previous study, inspired by frog skin, which is multifunctional, we created wrinkled slippery coatings by combining two processes: degradable diblock copolymer (diBCP) self-assembly [i.e., polystyrene-*b*-polylactide (PS-*b*-PLA)] and hydrolysis-driven dynamic release—induced surface wrinkling.^25^ Surfaces with microwrinkles are curved and resistant to biofouling. Gyroid-forming PS-*b*-PLA can be used to produce a nanoporous template with cocontinuous nanochannels; these nanochannels generate strong capillary forces that can trap and store infiltrated lubricants. We also fabricated slippery structural colloidal coatings by applying two layers of lubricant to low-surface-energy particulate films without the need for a complex hole-making process.^26^ These particulate films were composed of polytetrafluoroethylene or PDMS micelle dispersions and were created through spray coating. A thermally driven viscous surfactant was employed as a bottom adhesive primer that improved durability, and infused lubricant served as a top slippery layer. The sticky and fluid intrinsic properties of polymeric fluids with low-surface energy can eliminate polar interactions with biomolecules, making the removal of foulers easier.^24,27−30^ Our materials all exhibited remarkable surface-protective properties, including anticorrosion, antibiofouling, self-healing, anti-icing, and self-cleaning properties.^25,26^ Slippery liquid-infused porous surfaces are stable when submerged and do not degrade under a wide range of static environmental conditions. Unfortunately, marine foulers do accumulate on these surfaces as the lubricant is slowly depleted, and the lubricant is easily removed under hydrodynamic shear forces.^31−33^ This limits the applicability of these surfaces in dynamic applications. Besides, end-grafted PDMS brush approaches have been developed for antibiofouling, with their flexible polymer chains creating a dynamic surface that hinders the stable attachment of algal cells.^34,35^ Nevertheless, developing effective coatings that combine both low surface energy and geometric topography remains challenging; it requires balance among surface chemistry, size, geometry, and spatial feature arrangement to ensure the widest applicability.

**Figure 1:**
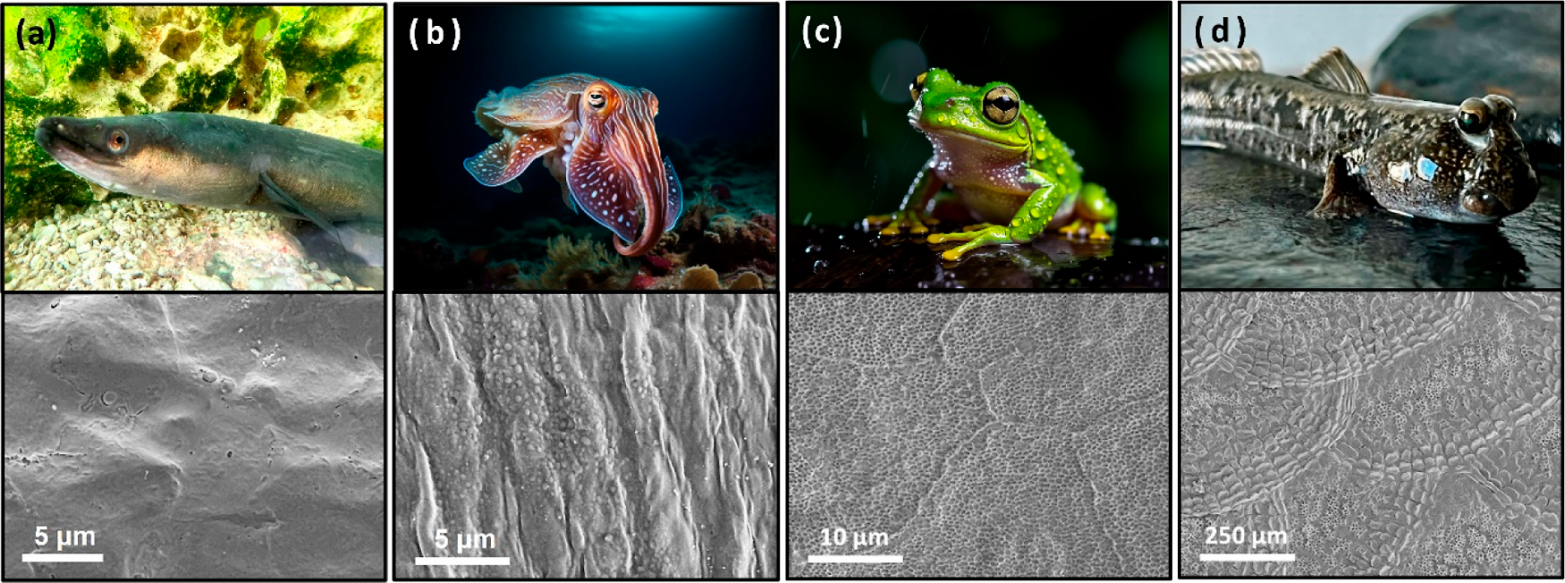
Examples of animals with slippery epidermis: (a) eel, (b) cuttlefish, (c) frog, and (d) mudskipper. Upper images represent living bodies, and lower images are surface of fresh and natural skin of each animal under scanning electron microscope (SEM). Illustrations of living bodies, including cuttlefish and frog, were generated using AI.

Several animals other than frogs have a slippery epidermis to counteract biofouling.^1,25^ For example, the outer layers of the eel,^36,37^ cuttlefish,^38^ frog,^25^ and mudskipper^39^ all have hierarchically organized morphologies and are covered with mucus or a lubricating fluid, regardless of whether their surface comprises scales or skin layers (Figure 1). Inspired by such animals with slippery epidermis, we successfully constructed durable and slippery antibiofouling coatings with liquid-like wetting buckled surfaces from silicon-containing self-assembled diBCP, where the buckling was induced through dynamic interfacial release. The core diBCP material was polystyrene-*block*-poly(dimethylsiloxane) (PS-*b*-PDMS). Because silicon-containing polymers have low surface energy,^19,40^ they can easily flow over and cover a surface after it has been subjected to controlled thermal treatment, generating a slippery wetting layer. Different from traditional slippery liquid-infused porous surfaces,^24−30^ the developed liquid-like PDMS wetting layer, chemically bonded with PS, provides stable slipperiness and does not flow away. To determine the influence of PDMS segment length on antibiofouling performance, test samples with various PDMS volume fractions were examined [PS47K-*b*-PDMS09K (PSDS-4709), PS30K-*b*-PDMS39K (PSDS-3039), and PS28K-*b*-PDMS85K (PSDS-2885)]. The surface characteristics of the samples—including their transparency and antibiofouling, anti-icing, and self-cleaning properties—were examined under various conditions. To the best of our knowledge, this is the first instance of the fabrication of silicon-containing copolymer-based liquid-like wetting buckled surfaces for use as durable slippery antibiofouling coatings. In particular, the ability to fabricate an ecofriendly silicon-based lubricant layer without needing to use fluorinated compounds and costly material precursors is an advantage in industrial practice. We believe that the proposed approach is up scalable for use in numerous practical applications, such as biomedical fluid handling, antibiofouling, fuel transport, self-cleaning windows, and optical devices.

## Results and Discussion

### Self-Assembled Morphology of Bulk PS-*b*-PDMS

**Scheme 1:**
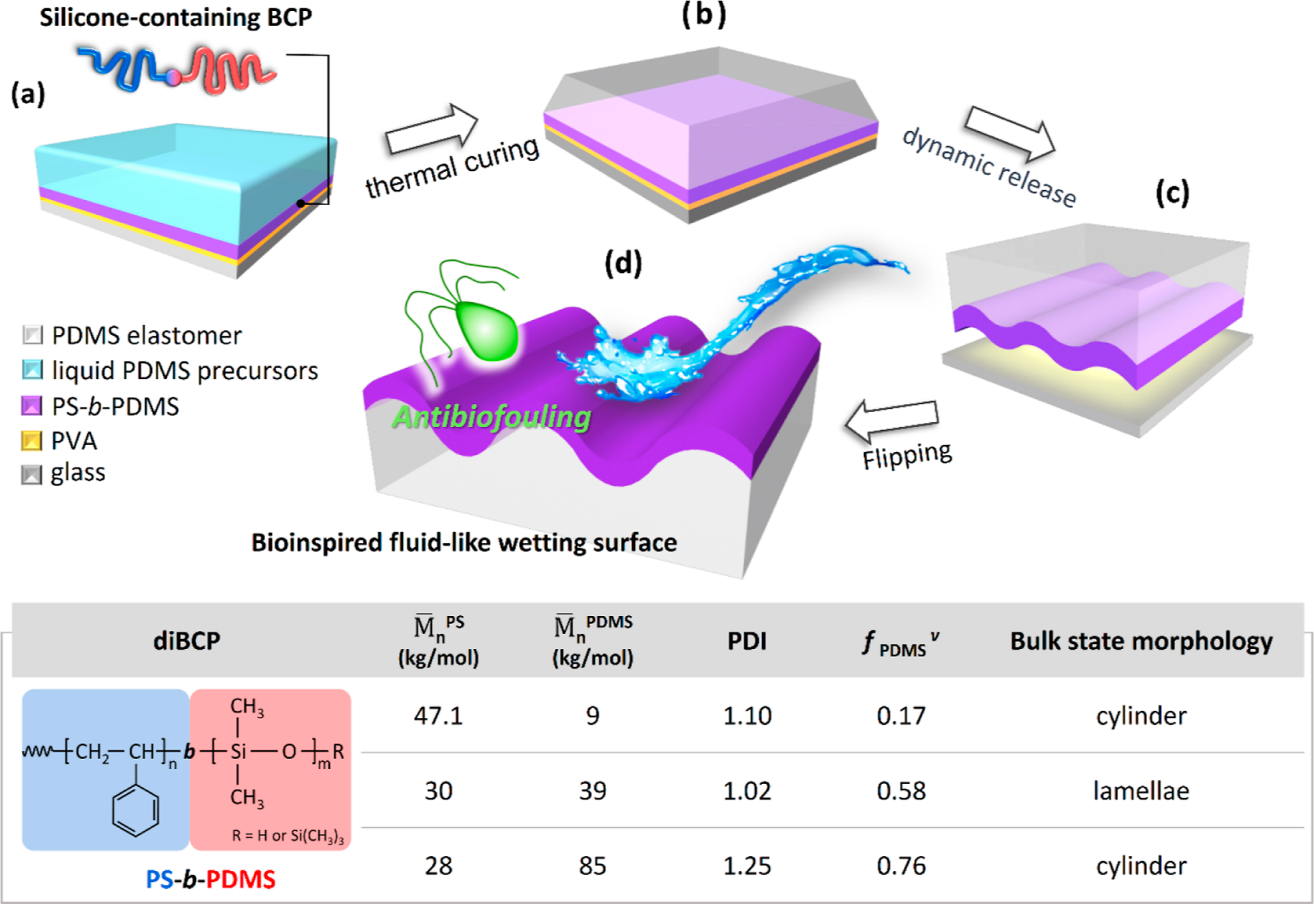
Illustration of Fabrication of Durable and Slippery Liquid-like Surface Composed of Silicon-Containing-diBCP-Derived Buckled Patterns for Antibiofouling (a) PVA and PS-*b*-PDMS solutions were spin-coated onto commercially available glass substrate in sequence, and liquid PDMS precursor was then cast. Sky blue, purple, yellow, and dark gray represent liquid PDMS precursor, PS-*b*-PDMS, PVA sacrificial layer, and glass substrate, respectively. (b) Thermal treatment was conducted to cure liquid PDMS, which formed crosslinked PDMS elastomers. (c) PVA sacrificial layer was dissolved using deionized water to release testing sample. (d) Flipping sample yielded micrometer-sized buckle pattern covered with annealed PS-*b*-PDMS to provide slippery liquid-like surface that can serve as stable and durable antibiofouling coating.

As mentioned, although various nature-inspired SLIPS antibiofouling coatings have been developed, the majority have the drawback of the lubricant flowing away over time under hydrodynamic shear forces. To solve this problem and extend the potential applications of SLIPSs, silicon-containing diBCP with a buckled pattern was fabricated and employed in stable slippery antibiofouling coatings. As illustrated in Scheme 1a, a water-soluble thin film comprising poly(vinyl alcohol) (PVA) was spin-coated onto a clean glass substrate; this film served as a sacrificial layer. A prepared polymer solution (pure PS or PS-*b*-PDMS diBCP solution) was then spin-coated onto the sacrificial layer to create a uniform and stiff polymer film that was used as the capping layer in the bilayer buckle system. Subsequently, a liquid PDMS precursor was cast onto the stiff polymer film, which was subsequently heated at 70 °C for several hours; this thermal curing enabled cross-linking the PDMS elastomer layer (Scheme 1b). The sample was then allowed to cool. To release the internal compressive load caused by the PDMS cross-linking and the contraction that occurred during cooling, the composite sample was immersed in deionized water to dissolve the sacrificial layer and lead to dynamic-interfacial-release-induced surface instability (Scheme 1c). Flipping the bilayer composite yielded a micrometer-sized buckled surface (Scheme 1d). Because silicon-containing polymers have low surface energy, they easily flow over and cover surfaces after they have undergone suitable thermal or solvent annealing, making the surfaces slippery. Three test samples with different PDMS volume fractions—PSDS-4709, PSDS-3039, and PSDS-2885—were used to determine the influence of PDMS segment length on antibiofouling performance. The molecular characteristics of the PS-*b*-PDMS samples—such as their molecular structural formula, volume fraction, molecular weight, polymer dispersity index, and microphase-separated structure—are listed in the table of Scheme 1. While controlling the self-assembly conditions, we fabricated micrometer-sized buckled patterns covered with PS-*b*-PDMS layers with various nanometer-sized self-assembled morphologies, including two-dimensional (2D) hexagonally packed cylinders and one-dimensional (1D) lamellae for further biofouling deposition examination.

**Figure 2:**
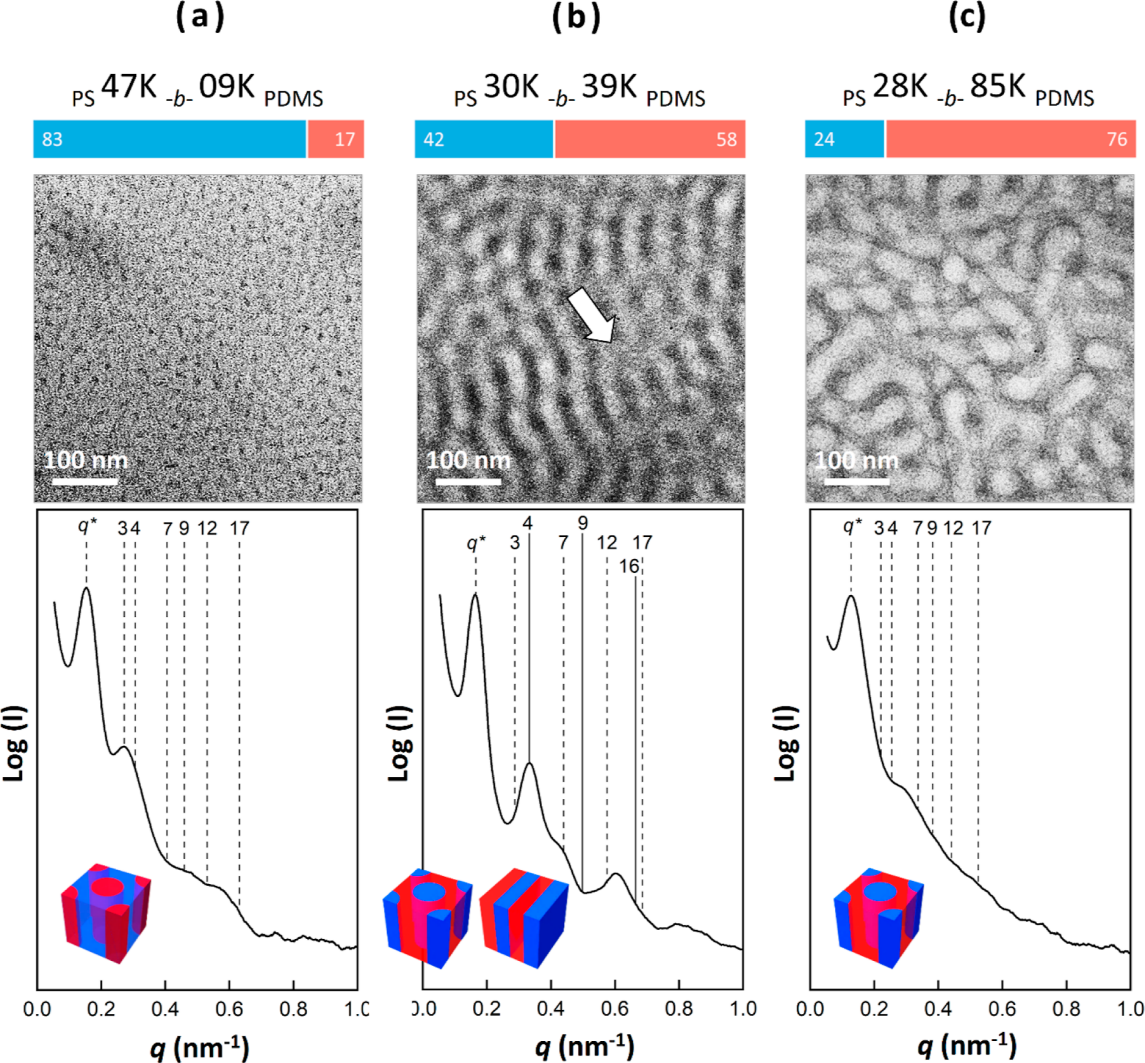
TEM images and corresponding 1D SAXS profiles of bulk-state self-assembled PS-*b*-PDMS diBCPs with various PDMS volume fractions: (a) PSDS-4709, (b) PSDS-3039, and (c) PSDS-2885. All samples were observed without use of staining agent. Upper blue and red bars indicate volume fractions of PS and PDMS, respectively. Inset: corresponding schematics of PSDS-4709 cylinder, PSDS-3039 containing coexisting lamellae and cylinders, and PSDS-2885 cylinder structures. In these representations, blue signifies PS segment, and red signifies PDMS segment.

First, the self-assembled morphology of the PS-*b*-PDMS samples had to be confirmed because their microphase separation structure, including their mechanical strength and the continuity of the slippery layers, is critical to their antibiofouling performance. The self-assembled structures of BCPs can be manipulated by adjusting their volume fractions and molecular weights. The molecular weights of the PSDS-4709, PSDS-3039, and PSDS-2885 samples were 56,000, 69,000, and 113,000 g/mol, respectively. The volume fractions of the PDMS blocks were calculated on the basis of assumed densities of 1.02 g/cm^3^ for PS and 0.97 g/cm^3^ for PDMS.^41^ Consequently, the volume fractions of the PDMS blocks in samples PSDS-4709, PSDS-3039, and PSDS-2885 were determined to be 17, 58, and 76%, respectively. Transmission electron microscopy (TEM) and small-angle X-ray scattering (SAXS) were used to identify the microphase separation structures of the three PS-*b*-PDMS diBCPs in their bulk state (Figure 2). To minimize solvent affinity between constituent blocks, we employed a nonselective and neutral solvent for casting the samples. The selected solvent was cyclohexane, which had a solubility parameter δ of 8.2, a value between the solubility parameters of PS and PDMS (9.1 and 7.4, respectively). To obtain self-assembled phases, cyclohexane-solution-cast PS-*b*-PDMS diBCPs were thermally annealed at 140 °C to eliminate their thermal history. Because of large mass—thickness contrast due to the high atomic number of silicon compared with those of carbon and hydrogen, the PS-*b*-PDMS diBCPs were examined under TEM observation without being stained. The PDMS microdomains appeared relatively dark in the TEM images, whereas the PS microdomains appeared bright. As shown in Figure 2a, because PDMS segments were a minor phase in the PSDS-4709 sample, they were observed as dark cylinders that were favorably dispersed in a bright matrix (i.e., PS domain). The corresponding 1D SAXS profile confirmed the existence of a cylinder phase with the space group p6 mm for which scattering peaks were found at the following q* ratios: 1:√3:√4:√7:√9:√12:√17. Therefore, on the basis of the TEM and SAXS results, the PSDS-4709 sample was identified as a typical cylindrical phase material composed of PDMS cylinders in a PS matrix.

For sample PSDS-3039, which had a higher volume fraction of PDMS segments, the TEM image contained slightly blurry regions between the cylinder-like domains (Figure 2b, white arrow). This was due to initiation of the fabrication of lamellar structures, which led to a reduction in local contrast in the TEM image. The coexistence of cylinder-like projections and slightly blurry sheet regions indicated the coexistence of cylinder and lamellar phases. The corresponding 1D SAXS profile contained coexisting reflection peaks at relative q* ratios of 1:2:3:4 (i.e., 1:√4:√9:√16) and 1:√3:√7:√12:√16:√17, further confirming the coexistence of cylinder and lamellar phases with the space groups p6 mm and pm, respectively. In the PSDS-2885 sample, which had the highest volume fraction of the PDMS segments, these segments constituted the majority of the testing sample such that in the TEM image, relatively bright worm-like domains (i.e., PS regions) appeared to be dispersed in the dark PDMS matrix (Figure 2c). PDMS has a low glass transition temperature (*T*_g_) and is thus soft and flowable under ambient conditions. This meant that the PSDS-2885 sample deformed easily, as reflected by the TEM image and SAXS result. In the SAXS signals, the peaks representing cylindrical structures could not be clearly identified. However, the ambiguous presence of the 1:√3:√4:√7 peaks still enabled us to identify the existence of cylinders. Our experimental results on the self-assembled morphologies of PS-*b*-PDMS samples with various PDMS volume fractions exhibited similarities to the symmetric phase diagram predicted by the Flory–Huggins theory. In addition, the repeating distance *L*_0_ calculated from SAXS is in the range of 37–48 nm for the PS-*b*-PDMS samples studied. Consequently, the thin film thickness measured is around 7–11 times larger than the repeating distance *L*_0_. This significant difference between the film thickness and *L*_0_ suggests that there should be enough space to develop a complete wetting layer on the surface without being affected by thickness confinement effects (see Table S1 for more details).

### Formation of Liquid-like Wetting Surfaces through Thermal Treatment

**Figure 3:**
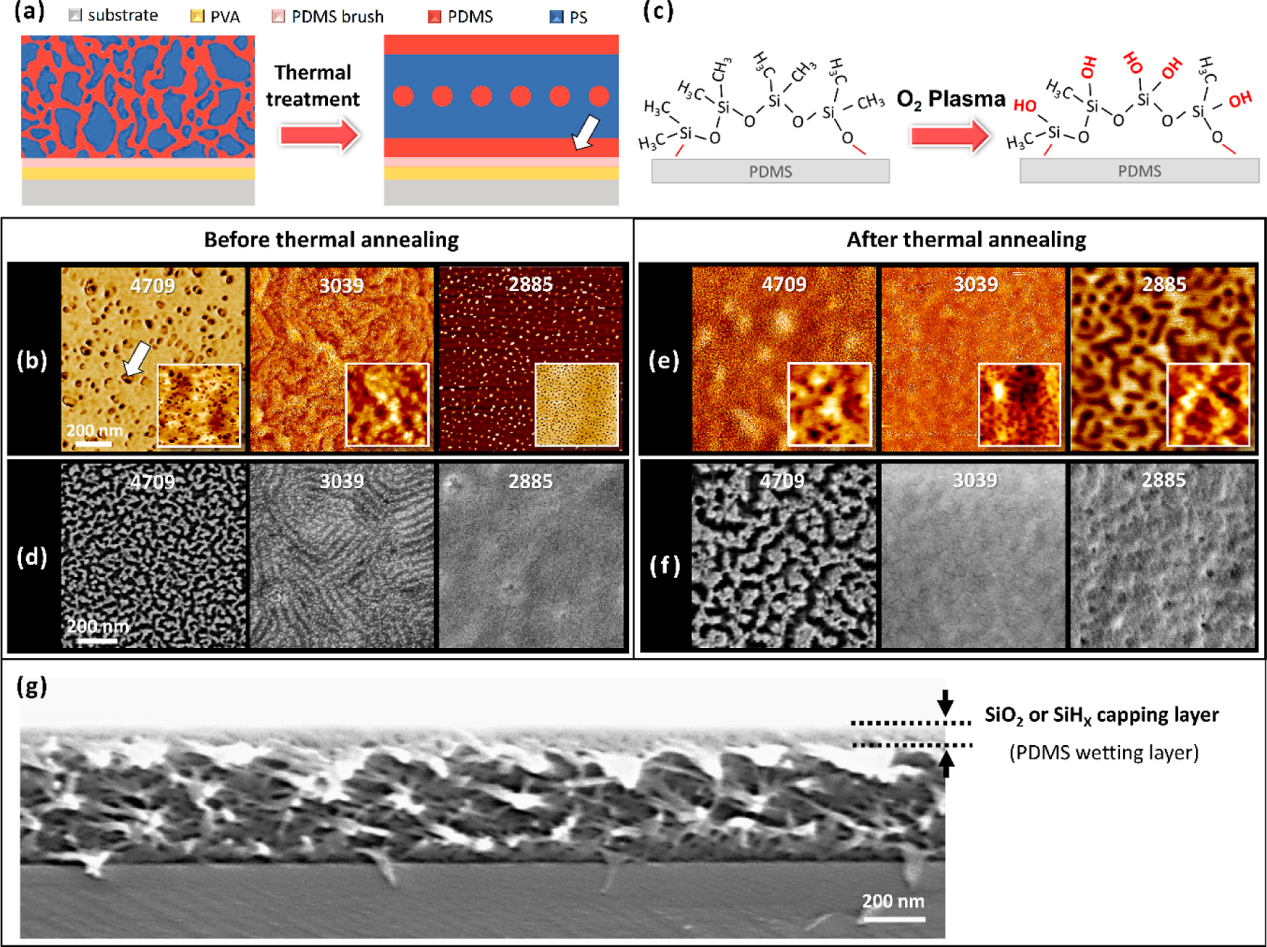
Self-assembled morphologies of interfaces of PS-*b*-PDMS thin films before and after thermal treatment. (a) Schematic of PS-*b*-PDMS layer after thermal treatment, which induced formation of PDMS wetting layers at interfaces with air and PDMS brushes. (b) AFM phase imaging results of self-assembled PS-*b*-PDMS thin films before thermal treatment. Inset: contrast topographic AFM images. (c) Schematic of formation of SiO_2_ or SiH_*X*_ layers from PDMS wetting layer after O_2_-RIE process. (d) SEM images of self-assembled PS-*b*-PDMS that had not undergone thermal treatment but had undergone O_2_-RIE. (e) AFM phase imaging results of self-assembled PS-*b*-PDMS thin films after thermal treatment. (f) SEM images of self-assembled PS-*b*-PDMS diBCPs that have undergone thermal treatment followed by O_2_-RIE. For each AFM image, scanning range was 1 × 1 μm^2^. (g) SEM cross-sectional view of PSDS-3039 after thermal treatment, followed by O_2_-RIE process, reveals the formation of a SiO_2_ or SiH_*X*_ capping layer on the sample surface.

The substrates were cleaned using ultrasonic solvent vibration, and then ultraviolet (UV)–ozone irradiation was performed to create a hydrophilic oxide layer on the surface of the substrates for PVA sacrificial layer deposition. After that, PDMS brushes were coated on the PVA layer for surface modification to create a PDMS-selected interface. Flat thin-film samples were obtained through spin-coating onto a substrate (e.g., silica or PDMS elastomers) modified with PDMS brushes. During the self-assembly process, some PDMS segments floated to the membrane interfaces due to their low surface energy, but most of the membrane interfaces comprised random PS and PDMS domains. PDMS is known to have low surface energy, and in an effort to reach a stable state, PDMS tends to move toward interfaces with low-energy barriers. Therefore, to cause liquid-like PDMS segments to self-assemble at the interfaces of the PS-*b*-PDMS diBCP thin films, thermal treatment was conducted to make the PDMS segments move to the interface layer with lower energy, such as the interface of air ambient surface and the brush-treated substrate surface; this resulted in the formation of a continuous PDMS wetting layer, as shown in Figure 3a. Note that the buckled PS-*b*-PDMS surfaces were fabricated using a dynamic-interfacial-release-induced surface instability approach (please refer to our previous research^42^). The sacrificial layer in a sample was dissolved such that the sample was released from the substrate, yielding a buckled PS-*b*-PDMS/PDMS composite. The buckled surface was the interface that was originally in contact with the PDMS brushes (as indicated by the white arrow in Figures 3a and S1) rather than the interface that was originally in contact with air. We investigated the effect of thermal treatment on the bottom layer of the self-assembled PS-*b*-PDMS (i.e., the interface that came into contact with the PDMS brushes). Atomic force microscopy (AFM) was conducted to identify the self-assembled morphology of the interface in contact with the PDMS brushes. The as-cast film thicknesses of the PSDS-4709, PSDS-3039, and PSDS-2885 samples were approximately 70, 110, and 220 nm, respectively. Differential scanning calorimetry (DSC) (Figure S2) revealed that the PS *T*_g_ of the three diBCP PS-*b*-PDMS samples was positively correlated with the molecular weight of the PS; its value was between 90 and 105 °C, whereas the *T*_g_ of PDMS is known to be approximately −125 °C.^43^ These results indicated that, at room temperature, the PS was in a glassy state, whereas the PDMS was in a fluid state. In the AFM images shown in Figure 3b, the brighter areas were attributable to the stiff PS matrix, whereas the darker areas corresponded to the softer PDMS domains. Dark elliptical PDMS regions (white arrow) appeared to be dispersed within the PS matrix (bright areas) in the AFM phase image of PSDS-4709, which was expected because PSDS-4709 comprised mostly PS. The contrast of a topographic AFM image is related to the roughness of the material depicted in the image. The insets of Figure 3b show slightly concave PDMS regions dispersed within the bright PS matrix. Due to the relatively small differences in height between the PS and PDMS regions, the AFM topography images did not have the same level of clarity as did the AFM phase images.

AFM is well-known to be generally suitable for measuring the surfaces of solid objects. However, measurements can be distorted when they are based on AFM images of surfaces coated with a sticky fluid, such as fluid PDMS. To gain deeper insight into the microstructures beneath the surface PDMS layer, oxygen reactive ion etching (O_2_-RIE) was employed to enable PS-*b*-PDMS diBCP patterning prior to scanning electron microscopy (SEM) observation.^43^ Because PS segments comprise C and H, they are converted into CO_2_ and H_2_O during oxygen plasma treatment, and the aforementioned molecules are then discharged by the RIE vacuum system. By contrast, PDMS comprises C, O, and Si; in the RIE process, the C in the organic compound is removed, and the other elements are employed to create SiO_2_ and SiH_*X*_ particles (Figure 3c). Thus, O_2_-RIE transforms liquid-like PDMS into solid SiO_2_ or SiH_*X*_ while simultaneously decomposing and removing PS segments, yielding a nanostructured inorganic pattern.^44^Figure 3d presents SEM images of the self-assembled PS-*b*-PDMS diBCPs that have not been thermally treated but that have undergone O_2_-RIE treatment, and these images show more cylindrical and worm-like structures than the structures observed through AFM. This indicated that the PDMS domains, which were originally encapsulated by PS, were transformed into SiO_2_ and exposed, making them easier to see. Due to spatial confinement affecting the self-assembly of diBCP into thin film, the self-assembly patterns were slightly different from those in the bulk material, taking the form of deformed worm-like structures instead of classical cylinders. As expected, increasing the volume fraction of PDMS in the PS-*b*-PDMS resulted in an increase in the thickness of the PDMS wetting layer on the thin film’s surface. For example, after O_2_-RIE treatment, PSDS-3039 exhibited an intermingled SiO_2_ structure comprising both sheets and worm-like patterns. Conversely, PSDS-2885 had a larger amount of PDMS on its surface, and this PDMS had nearly continuous SiO_2_ sheet-like morphology after O_2_-RIE treatment; only a few micropores remained from the decomposition of PS domains.

Figure 3e displays AFM images of the test samples after they had undergone thermal treatment (70 °C for 1 h). The self-assembled microstructures were clearly more distinct after the thermal treatment. This was because the thermal treatment provided energy to the polymer chains, enabling them to self-assemble more effectively, resulting in a structure that was more well-defined. However, although the thermal treatment enhanced the chains’ self-assembly ability, it also provided more opportunities for PDMS to come to the film surface and form a wetting layer, which could not be detected through AFM. The thermally treated test samples with a PDMS wetting layer could be identified more clearly after O_2_-RIE treatment, as shown in Figure 3f. After the thermal treatment, larger and more numerous SiO_2_ island structures could be observed on the PSDS-4709 sample, indicating the formation of a greater number of PDMS wetting domains. However, because PS was the major phase in PSDS-4709, the PDMS domains had low mobility, which prevented the formation of a large-scale wetting layer. The PSDS-3039 sample exhibited even more pronounced changes, transitioning from coexisting sheet-like and worm-like structures before thermal treatment to a continuous SiO_2_ layer after thermal treatment. Corresponding SEM cross-section results showed that a SiO_2_ or SiH_*X*_ capping layer (originally PDMS wetting layer) could be clearly observed on top of the nanostructured SiO_2_ or SiH_*X*_. The total film thickness was approximately 410 nm, with the PDMS capping wetting layer thickness around 50–80 nm (Figure 3g). For the PSDS-2885 sample, the thermal treatment had a weaker effect. Because the PSDS-2885 sample contained mostly PDMS, it had sufficient flowability to form a wetting layer during the self-assembly process at room temperature; additional thermal treatment was not required. The chemical composition of the O_2_-RIE-treated PS-*b*-PDMS samples was further characterized using X-ray photoelectron spectroscopy (XPS). Figure S3 displays the XPS signals of the three PS-*b*-PDMS films before and after thermal treatment following O_2_-RIE treatment. Clear Si_2p_, Si_2p_, C_1s_, and O_1s_ peaks were detected at 102, 153, 285, and 530 eV, respectively. The intensities of the peaks at 102 and 530 eV were partly attributable to the intrinsic SiO_2_ layer on the Si wafer. The elemental composition of PDMS includes Si, C, O, and H; most of the C detected in the PS-*b*-PDMS samples was present in the PS on the surfaces of the samples. Therefore, the change in the proportion of C was an important basis for making judgments in the XPS detection. During thermal treatment, the PDMS chains were thermally driven to the interfaces, and the proportion of PS on the surface decreased, resulting in a decrease in the intensity of the C_1s_ peak. Additionally, with this reduction in the amount of C, more O became accessible to Si. The increase in the intensity of the O_1s_ peak (i.e., the conversion of PDMS polymeric chains into SiO_2_) indirectly indicated that after the thermal treatment, the PDMS content on the surfaces was higher than that before the thermal treatment. To ensure that a sufficient PDMS wetting layer formed on the surface of the samples used in antibiofouling testing, thermal treatment was applied to all these samples.

### Buckled PS-*b*-PDMS with Liquid-like Wetting Layer as an Antibiofouling Coating

**Figure 4:**
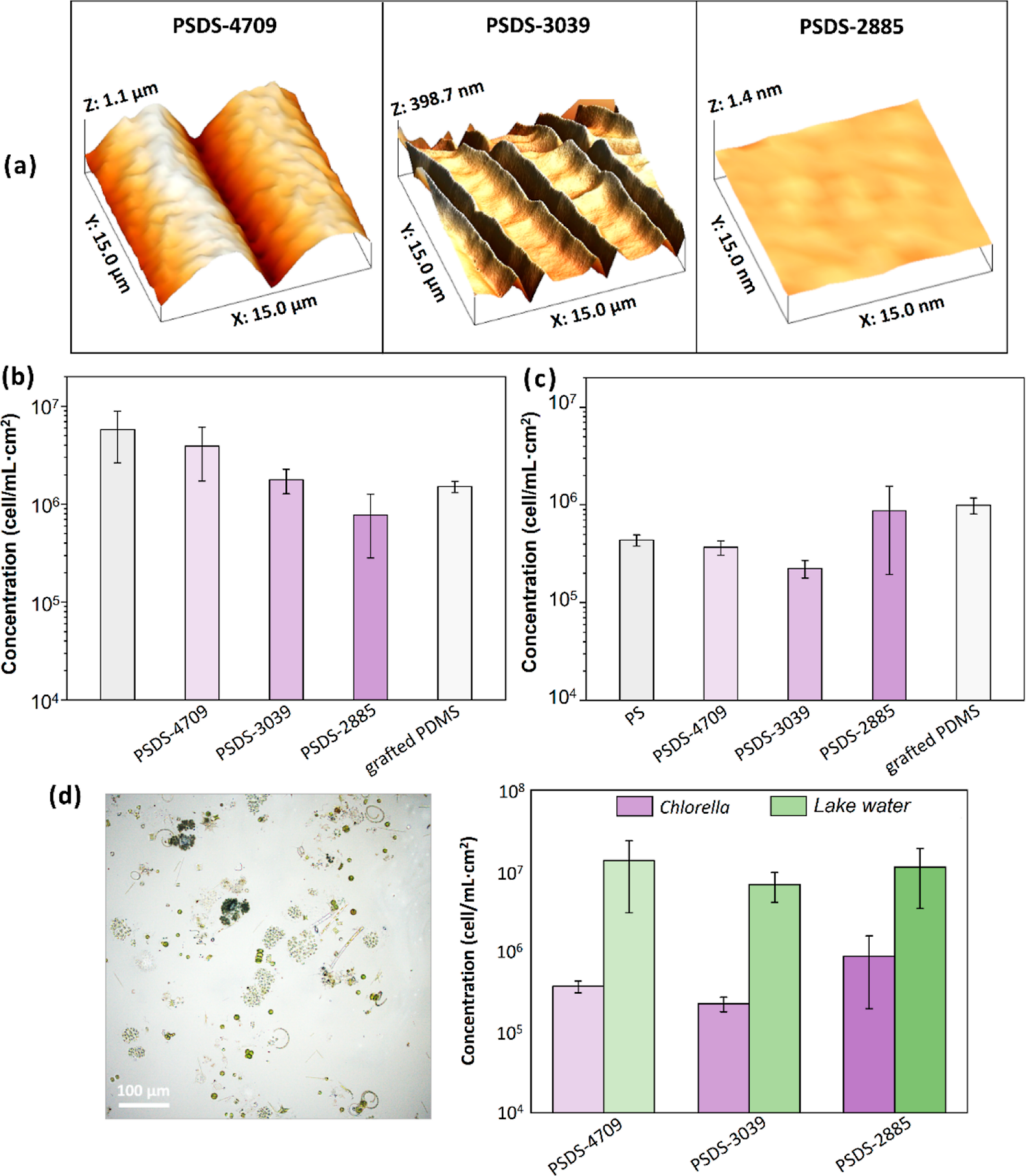
BCP wrinkling structure fabricated using hydrolysis-driven dynamic-release-induced process. (a) Morphologies of dynamic-released wrinkled BCPs, which made up stiff layer. Comparison of green algae settlement on (b) flat film and (c) buckled samples with various PDMS components. (d) Optical microscopy image of microalgae from natural lake water (left) and comparison of green algae (*Chlorella*) and microalgae from lake water settled on bulk samples with various PDMS components (right).

Dynamic-interfacial-release-induced surface instability created buckled surfaces of the PS-*b*-PDMS/PDMS bilayer composites owing to the generation of external stress caused by cross-linking-induced volumetric shrinkage of PDMS. According to our previous study,^45^ the geometric parameters of diBCP bilayer wrinkles, including their wavelength and amplitude, are affected by the thickness of the capping layer and the mechanical strength of the bilayer’s components (i.e., the elastic moduli of the diBCP thin film and PDMS substrate in this study). That is, the elastic moduli of the three diBCPs would affect the morphologies of generated buckles because of the different PDMS volume fractions of each sample. Figure 4a displays three-dimensional AFM morphological images of liquid-like surfaces composed of the three PS-*b*-PDMS diBCPs covering PDMS elastomers (the mixing ratios of liquid PDMS precursor/cross-linker 20:1 by weight). As mentioned, all the test samples were subjected to thermal treatment to create a PDMS wetting layer. The theory behind the mechanism of buckle formation indicates that the mechanical strength of the capping layer must differ considerably from that of the substrate if a buckle pattern is to be generated. For PSDS-4709, PS was the primary component of the capping layer, which was much harder than the PDMS elastomers. As anticipated, a distinct wrinkle pattern formed after the buckling process. The wavelength and amplitude of the wrinkles were approximately 10 μm and 500 nm, respectively. When the volume fraction of PDMS was higher, the capping layer was softer. Consequently, the typical wrinkles were not generated in the PSDS-3039 sample; instead, period-double and wrinkle morphologies coformed. The coexistence of these two morphologies usually occurs when a composite with a thin or soft capping layer experiences high strain.^46^ Moreover, different from PSDS-4709 and PSDS-3039, PSDS-2885 had a flat surface without any buckled structures. To further confirm whether the buckles disappeared instead of being covered by the PDMS wetting layer, the samples were dipped into hydrogen fluoride to remove the PDMS wetting layer on the surfaces. As shown in Figure S4, PSDS-4709 and PSDS-3039 maintained their buckled surfaces after the removal of the PDMS wetting layer. In contrast, PSDS-2885 exhibited a flat surface without any buckles. This clearly supported our statement: because PDMS was the predominant component of PSDS-2885, the capping layer was highly fluid and lacked the mechanical strength necessary to induce buckle formation.

The geometric topography and surface chemistry of interfaces have crucial effects on how biofouling coatings interact with, settle on, and adhere to a surface. Surfaces with microbuckled patterns are curved and can resist biofouling because of their surface geometry. Low-surface-energy materials (e.g., PDMS) can eliminate polar interactions between biomolecules and surfaces, facilitating the removal of foulers.^25,26^ To demonstrate the antibiofouling performance of the liquid-like buckled PS-*b*-PDMS surfaces, a typical green alga species, *Chlorella* sp. DT, was used as a model organism. Figure 4b displays the average coverage of green alga on nonbuckled surfaces of PS, PSDS-4709, PSDS-3039, and PSDS-2885 samples. To compare our strategy with strategies involving surface grafting for the formation of a liquid-like surface to prevent biofouling, we prepared PDMS-grafted surface by grafting a PDMS–Cl polymer brush on a PDMS substrate. The molecular weight of the PDMS–Cl used for PDMS grafting was approximately 3000 g/mol. Because no geometric topography effects and no low-surface-energy fluids were exerted on the PS film, the green alga easily settled onto the surface of the PS film. The average coverage was approximately 5.76 × 10^6^ cells/mL cm^2^, which was the highest algal coverage of all the samples tested. By contrast, the test samples covered with a liquid-like PDMS layer exhibited relatively low algal coverage. The residual green algal coverage values of the flat PSDS-4709, PSDS-3039, and PSDS-2885 surfaces were 3.92 × 10^6^, 1.78 × 10^6^, and 7.73 × 10^5^ cells/mL cm^2^, respectively. Clearly, the antibiofouling performance was higher when the PDMS chains in the PS-*b*-PDMS diBCP were longer, and the PSDS-2885 film exhibited remarkable antibiofouling performance in this experiment. When the PDMS chains were longer, the liquid-like surface exhibited higher instability and fluidity, which helped prevent early stage adhesion of the biofouling coatings. In addition, the PDMS-grafted PDMS also had remarkable antibiofouling performance; the corresponding residual green algal coverage was 1.39 × 10^6^ cells/mL cm^2^. According to the biofouling coverage results, the antibiofouling performance of the PDMS-grafted PDMS was between those of PSDS-3039 and PSDS-2885.

The test film samples were structuralized to create buckled surfaces for investigating the influence of geometric topography on the antibiofouling ability of the liquid-like surfaces. Figure 4c displays the average green algal coverages of the prepared film surfaces after surface buckling. The buckle pattern was indeed discovered to greatly enhance the antibiofouling performance of the samples (the buckled PS, PSDS-4709, and PSDS-3039) regardless of whether their surfaces had a liquid-like layer. Their coverage values of residual green alga were 4.37 × 10^5^, 3.69 × 10^5^, and 2.25 × 10^5^ cells/mL cm^2^, respectively. Interestingly, the buckled PSDS-3039 sample exhibited the highest antibiofouling performance of the five samples, even though PSDS-2885 had a higher PDMS volume fraction (i.e., a thicker liquid-like surface layer). This was because, as mentioned, a high proportion of PSDS-2885 was PDMS, meaning that the material had insufficient mechanical strength to enable the formation of buckles; thus, its antibiofouling performance was not enhanced by geometric topography. A similar result was obtained for the PDMS-grafted PDMS, namely, a flat slippery film without topographical effect. Therefore, its antibiofouling performance before and after buckling was similar; in both these cases, the material behaved like a flat liquid-like film (comparison of Figure 4b with 4c). The coating with a liquid-like surface layer and structure, namely buckled PSDS-3039 with a liquid-like PDMS wetting layer, exhibited the most stable antibiofouling effect. The corresponding laser-scanning confocal microscopy images of the surfaces, including buckled PS, PSDS-4709, PSDS-3039, PSDS-2885, and grafted PDMS surfaces, after biofoulers deposition were shown in Figure S5. Additionally, diverse and complex organisms from natural environments were used to closely simulate real-world environments. We collected samples from lake water, filtered, and concentrated them to obtain a high-concentration mixed algal solution, and performed tests under controlled laboratory conditions. It yielded the same trend as using a single algal species (*Chlorella*): longer-chain PDMS exhibited better antifouling performance (Figure 4d). According to the mechanisms of fouling resist and antifouling, the PS-*b*-PDMS-driven antibiofouling coatings are more inclined toward the fouling resist mechanism, utilizing surface structure and unstable interface characteristics to resist algae. About surface brush grafting, it was influenced by the molecular length, shape, and density of the graft on the substrate. Although the PDMS-grafting approach also resulted in a liquid-like surface with antibiofouling ability, grafting sites were required on the surface. The number and position of grafting sites were influenced by the structure of the grafting molecules, spatial hindrance, and polarity. Herein, the grafting density of PDMS brushes on the substrate was determined to be 1.81 × 10^18^ molecules/cm^2^ (see details in Figure S6). The PDMS brushes were composed of particle islands, and their average roughness was around 0.525 nm (from AFM result). Clearly, the grafting sites were spaced widely and thus not conducive to a high grafting density. In other words, a continuous and complete liquid-like surface was more likely to form on the diBCP-based films. Most importantly, the PS-*b*-PDMS diBCPs had sufficient mechanical strength for surface buckling to occur, and their structured surfaces further enhanced their antibiofouling capability; such an enhancement could not be achieved through the brush-grafting approach.

### Other Properties, Applications, and Developments

**Figure 5:**
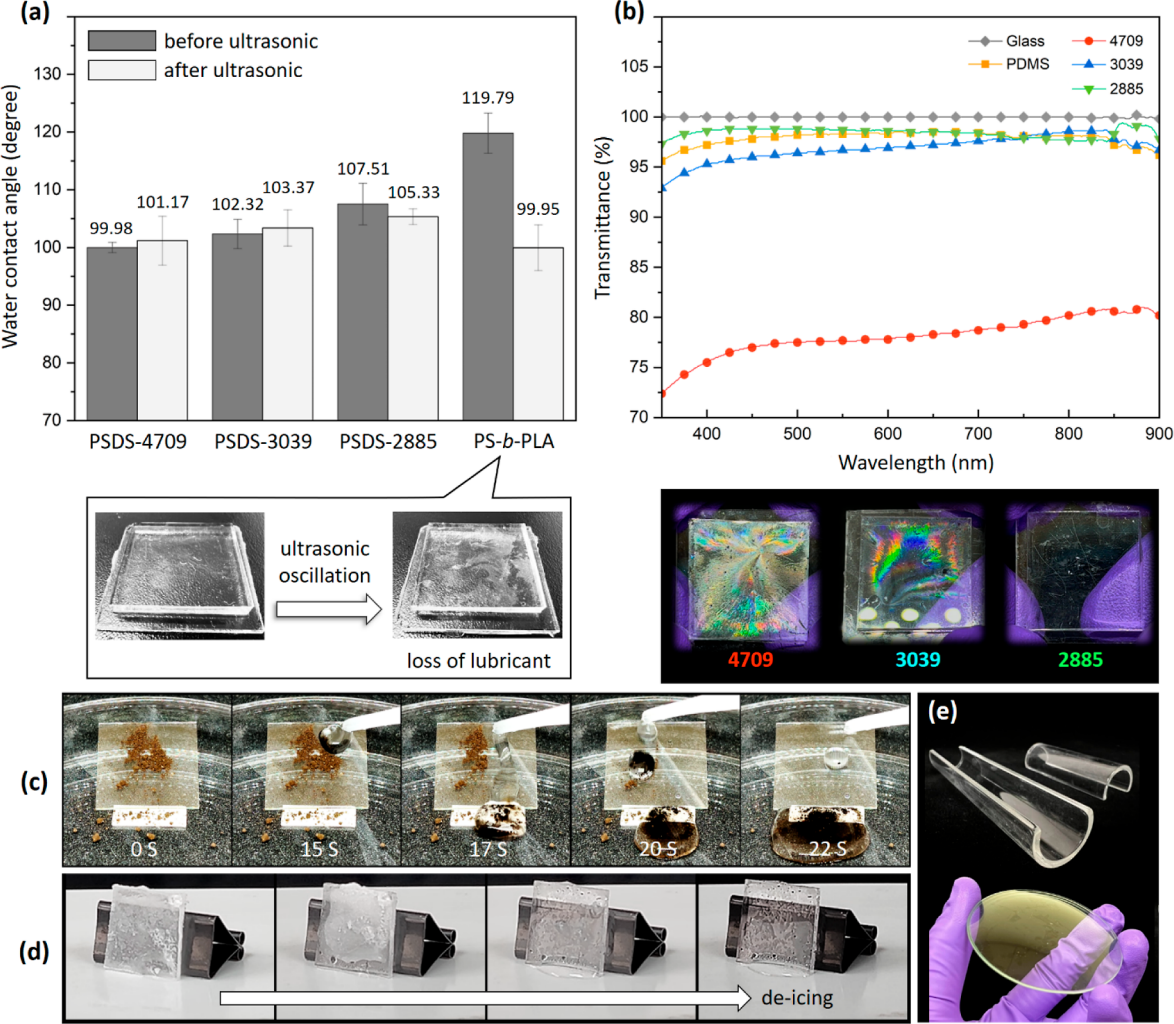
(a) WCAs for different samples after their resonance in ultrasonic bath for 30 min. (b) Transmittance spectra of glass, pure PDMS substrate, and buckled PS-*b*-PDMS surfaces. (c) Photographs of dirt particles on PS-*b*-PDMS samples to demonstrate their self-cleaning abilities. (d) Stills extracted from video showing composite’s ability to prevent initiation of ice nucleation and attachment of ice and frost on the surface. (e) Photographs of PSDS-2885 coatings on glass architectures of different shapes.

SLIPS antibiofouling coatings are unstable because the lubricants change over time and external forces affect the SLIPS system. This study’s buckled PS-*b*-PDMS films with liquid-like PDMS segments linked by covalent bonds on a solid surface should solve the problem of lubricant dissipation and other issues. To assess the reliability and stability of the buckled PS-*b*-PDMS films, test samples were subjected to vibration in an ultrasonic bath for 1 h. The results were then compared with those for the lubricant-infused nanoporous gyroid wrinkled PS developed in our previous study (Figure S7).^25^ To quantify the damaging energy transferred to the sample surface through ultrasonic oscillation,^47−49^ we employed the formula *Q* = *m* × *C*_*p*_ × Δ*T*, where *Q* is the energy (J), *m* is the mass (kg), *C*_*p*_ is the specific heat capacity (J/kg °C), and Δ*T* is the temperature change (°C). The damaging energy transmitted to the samples through ultrasonic oscillation was calculated to be approximately 84 kJ. This energy is equivalent to the force exerted continuously for 90 consecutive days by seawater on a bottle when the seawater is moving at a flow rate of 0.5 m/s (similar to the tidal speed of the Kuroshio Current, which passes Taiwan^50^). Changes in the contact angle of water droplets were used to confirm the stability of surfaces and thus the slipperiness of the surfaces (Figures 5a and S8). Before ultrasonic oscillation, the water contact angles (WCAs) of the samples increased with the increase of PDMS content due to the intrinsic hydrophobic properties of PDMS. Conversely, the sliding angles (SAs) decreased with the increase of PDMS content. This is because a higher PDMS content provides flatter and smoother surfaces, which facilitate the rolling of water droplets. In addition, the slippery buckled PS-*b*-PLA showed the lowest contact angle hysteresis (CAH) because it was covered with a thick lubricant layer, giving a relatively homogeneous and flat surface. In contrast, PSDS-4709, with the lowest PDMS content and a buckled surface, exhibited the highest CAH. After ultrasonic oscillation, for PS-*b*-PDMS coatings, the WCAs of the samples were similar to their original states without significant changes, indicating that our coatings possess stable properties and are not destroyed by ultrasonic agitation. Interestingly, their CAH decreased after ultrasonic agitation. We hypothesize that the ultrasonic agitation made the PDMS wetting layers more homogeneous, leading to lower CAH. By contrast, the slippery buckled PS-*b*-PLA not only showed a decrease in WCA but also an increase in CAH after ultrasonic agitation, indicating a loss of lubricating oil and an inability to provide reliable antibiofouling performance. The photographs in Figure 5a depict the nanoporous gyroid SLIPS (i.e., lubricant within the nanoporous gyroid PS-*b*-PLA template) before and after ultrasonic oscillation. The nanoporous gyroid SLIPS transitioned from having a clear and smooth appearance to appearing blurry and indistinct, indicating a considerable loss of lubricant and a reduction in the surface’s smoothness.

For additional chemical and physical resistance tests, we take buckled PSDS-3039 as an example due to its superior antibiofouling performance. In the chemical durability test, samples were immersed in 1 M HCl, 1 M NaOH, and seawater for 24 h, and their WCA were measured after gentle washing (Figure S9a). The results showed that the WCAs were not significantly affected by these chemical solutions, and their microstructures remained intact, indicating sufficient chemical resistance for practical applications. Although the WCA and surface morphologies were destroyed after 12 h of short-wavelength UV exposure, the coatings are not expected to be exposed to such high-energy UV light in normal use environments, such as seawater. Furthermore, samples were subjected to compressive stress tests to observe potential physical damage, as shown in Figure S6b. After applying a constant force of 0.6 kg W/cm^2^, deformation of the buckled patterns occurred on the surface, as indicated by the white arrow. Nevertheless, this stress level should be able to withstand most physical impacts encountered in aquatic environments and resist most situations in daily life.

To assess the feasibility of using the prepared liquid-like PS-*b*-PDMS films as antibiofouling coatings for underwater optical instruments, such as sensors and windows, UV–visible spectra of the films were obtained and compared with those of pure glass and pure PDMS substrates (Figure 5b). The glass substrate was employed as the benchmark, with its transmittance rate considered to be 100% under atmospheric conditions. Similar to the glass substrate, the pure PDMS substrate was highly transparent in the visible light range. By contrast, the PS-*b*-PDMS films with buckled patterns (PSDS-4709 and PSDS-3039 samples) were less transparent due to phenomena such as reflections by photonic crystals on the surface (as shown in the insets of Figure 5b and Movie S1). The PSDS-2885 sample, which did not exhibit obvious buckling structures, was the most transparent of the three PS-*b*-PDMS films. Another challenging aspect of slippery surfaces is the effect of deposited solid dirt. When solid particles come into contact with a slippery surface, they tend to roll into the lubricating layer, thereby compromising the layer’s antibiofouling performance. In this study, PDMS blocks serving as the liquid-like wetting layer were not present in the form of a liquid-phase oil. Thus, deposited dust particles could not sink into and become trapped within the layer. As shown in Figure 5c and Movie S2, no residual powder or pollutants remained on the liquid-like PS-*b*-PDMS surfaces after the water on the surfaces was flushed. Moreover, the anti-icing performance of the buckled PS-*b*-PDMS films was examined. The buckled PS-*b*-PDMS films (e.g., buckled PSDS-3039) were initially immersed in liquid nitrogen for 1 min to simulate freezing conditions (Figure 5d). Subsequently, the samples were removed and then generously sprayed with a water mist at room temperature, which resulted in the accumulation of frost and ice on their surfaces. Ultimately, the frozen samples were heated from room temperature to 100 °C for 3 min to facilitate the deicing process. Due to their low surface energy and liquid-like surface properties, the buckled PS-*b*-PDMS films exhibited remarkable anti-icing performance; ice nucleation did not occur on these surfaces, and a smaller amount of ice and frost developed on them (Movie S2). Because the films all comprised resilient polymers, the PS-*b*-PDMS films have ample mechanical strength to withstand abrupt and extreme temperature fluctuations. They can also be easily applied to surfaces of various shapes, such as curved surfaces and tubes (Figure 5e). They can be shaped to meet specific requirements for specific applications. This adaptability means that they have a wide scope of application.

## Conclusions

In this study, we created durable slippery antibiofouling coatings with liquid-like wetting buckled surfaces. The coatings were successfully constructed by combining dynamic-interfacial-release-induced buckling and self-assembled silicon-containing diBCP. The core diBCP material was PS-*b*-PDMS. Because silicon-containing polymers have low surface energy, they easily flow over and cover the surface of a material after controlled thermal treatment and thereby generate a slippery liquid-like wetting layer. Additionally, use of slippery polymeric fluids with low surface energy eliminates polar interactions with biomolecules, making the removal of biofouling agents easier. Furthermore, microbuckled patterns result in curved surfaces, which offer fewer points at which organisms can attach themselves to the structural topography. PS-*b*-PDMS diBCPs with various PDMS volume fractions were studied to determine the influence of PDMS segment length on antibiofouling performance. The experimental results demonstrated surfaces with buckled patterns had greatly enhanced antibiofouling performance, regardless of whether the surfaces were covered with a liquid-like layer. Furthermore, when the PDMS chain segments were longer (i.e., higher PDMS volume fraction), the wetting layer that formed was thicker and more complete, resulting in higher antibiofouling performance. Antibiofouling behavior was not correlated with the self-assembled microstructure of the PS-*b*-PDMS; thus, the polydispersity index of polymers does not need to be finely controlled during synthesis. This helps reduce the technical requirements and costs associated with the synthesis of BCPs. When the proportion of PDMS was too high, buckled patterns did not form because the material had insufficient mechanical strength, and the lack of these patterns led to poor antibiofouling capability. For optimal antibiofouling performance, we recommend selecting buckled PSDS-3039; this material combines geometrical effects with a low-surface-energy liquid-like surface. However, if the coating is intended for an underwater optical device, PSDS-2885 is the most suitable material. Although it does not have geometrical effects that would enhance its performance, it contains the longest flexible PDMS chains and is highly transparent.

Comparing with other research group’s results is important, but it is not easy because the experimental parameters of antibiofouling testing are not standardized. For example, the type of algae, the settlement environment, the flow rate, and the settlement time all vary, making it difficult to establish uniform standards for comparison. Despite these challenges, we attempt to compare various buckled samples using our own algae type and environment, including buckled PSDS-3039, buckled PS, buckled PDMS-grafted PDMS, and buckled gyroid surface infused with silicone oil (i.e., SLIPS-PDMS).^25^ As shown in Figure S10, buckled SLIPS-PDMS exhibited the best antibiofouling performance, due to their relatively thick lubricant coating layers. There was minimal remaining green algae coverage on their surfaces. However, as mentioned above, lubricant oils on the buckled SLIPS-PDMS surface easily flowed away under hydrodynamic shear forces (Figure 5a). In contrast, the PS-*b*-PDMS-driven coatings demonstrated robust characteristics even under ultrasonic vibrations. Therefore, the PS-*b*-PDMS-driven coatings should be considered an ideal candidate for antibiofouling applications compared to other surfaces. Moreover, the surfaces were found to be flexible, stable, transparent, and self-cleaning and to have easily tunable antibiofouling and anti-icing characteristics. Being able to fabricate an ecofriendly silicon-based lubricant layer without the need for fluorinated compounds and costly material precursors is an advantage in industrial practice. This advantage can be implemented in various applications. We believe that the proposed approach is up scalable for use in several practical applications, such as biomedical fluid handling, antibiofouling, fuel transport, anti-icing, self-cleaning windows, and optical devices.

## Experimental Section

### Materials

Wetland animal specimens were collected from mountainous regions and coastal areas of Taiwan. A liquid PDMS mixture was prepared using the Dow Corning Sylgard 184 elastomer kit in a mixing ratio of 20 parts liquid PDMS precursor to 1 part cross-linker. PVA with a molecular weight range of 118,000–124,000 g/mol was obtained from First Chemical Works and used as received. The PVA was dissolved in deionized water at a concentration of 1 wt %. Three diBCPs—PSDS-4709, PSDS-3039, and PSDS-2885—were acquired from Polymer Source; their molecular weights were 56,100, 69,000, and 113,000 g/mol, respectively. In our calculations of the volume fraction of PDMS in the microphase separation process, we assumed the densities of PS and PDMS to be 1.02 and 0.965 g/cm^3^, respectively.^41^ Cl-terminated PDMS brushes (average molecular weight ≈3000 g/mol) was purchased from Sigma-Aldrich and used without further purification for surface grafting. PS with a molecular weight of 260,000 g/mol was used as received to fabricate the stiff capping layer of bilayer buckled composites. Acetone and alcohol were employed as washing solvents and were used without purification.

### Fabrication of Buckled Composites and Liquid-like Surfaces

First, glass slides were washed with acetone and methanol to ensure they had clean surfaces. Afterward, to create a polymeric sacrificial layer, the PVA solution mentioned in the section of Materials was spin-coated onto a UV–ozone-treated glass slide at 1500 rpm for 30 s. Subsequently, a polymer (e.g., PS) thin film with a thickness of approximately 250 nm was prepared on the glass slides with PVA layer by spin-coating 1.5 wt % polymer solution at 1500 rpm for 30 s. For preparation of PS-*b*-PDMS thin films, PDMS brushes were coated on the PVA sacrificial layer to create a PDMS-selected interface for formation of a PDMS wetting layer. Toluene and cyclohexane were used as solvents to dissolve PS and PS-*b*-PDMS, respectively. The mixing ratios of the liquid PDMS precursor/cross-linker using a 20:1 weight ratio. A liquid PDMS mixture (30 g) was placed on the prepared PS or PS-*b*-PDMS thin film in a plastic Petri dish (10 × 10 cm^2^) and degassed under a moderate vacuum for 40 min. The resultant multilayer composite was placed in an oven for overnight thermal curing at 70 °C to cross-link the liquid PDMS mixture. A PDMS wetting layer was generated on the PS-*b*-PDMS thin film during the heating process and covered the surface of the sample. This composite was then cooled to room temperature to generate an elastomeric substrate. The thickness of the prepared PDMS layer was approximately 3 mm. To obtain a microbuckle structure, the PS/PDMS or PS-*b*-PDMS/PDMS bilayer composite was immersed in deionized water at approximately 35 °C for 3 h. The dissolution of the sacrificial (PVA) layer resulted in the release of the composite sample and spontaneous formation of buckles on the surface of the sample. This method relied upon the kinetic release of local strain, enabled by the greater dissolution of the sacrificial layer along the edges of the multilayer composite. Thermally induced cross-linking caused volumetric shrinkage of the composite, driving the development and stabilization of surface wrinkles.^25,42,45^ Eventually, buckled PS/PDMS composites and buckled PS-*b*-PDMS/PDMS composites with PDMS wetting layers were generated and employed in antibiofouling performance tests. In addition, PDMS was grafted onto a glass substrate by immersing clean UV–ozone-irradiated glass in PDMS–Cl solution for 1 h, and then the surface was rinsed with *n*-hexane to remove the polymer brush that had not grafted to the substrate. Finally, the surface was air-dried with nitrogen, thereby completing the preparation of the glass substrate with PDMS brushed on the surface.

### Alga Settlement and Adhesion Assays

The green alga *Chlorella* sp. DT was isolated from the dry surface of a power-transmitting cable on a mountain in central Taiwan.^51^ Algae were routinely cultivated in 250 mL flasks with sponge plugs containing 100 mL of *Chlorella* medium [10 mM KNO_3_, 1 mM MgSO_4_·7H_2_O, 0.5 mM Na_2_HPO_4_, 4.5 mM NaH_2_PO_4_·H_2_O, 20 μM CaCl_2_·2H_2_O, 50 μM FeSO_4_·7H_2_O, 50 μM EDTA·Na_2_·2H_2_O, 1 μM H_3_BO_3_, 1 μM MnSO_4_·H_2_O, 1 μM ZnSO_4_·7H_2_O, 0.01 μM CuSO_4_.·5H_2_O, and 0.01 μM (NH_4_)_6_Mo_7_O_24_·4H_2_O (pH 6.8)] to which was added 0.25% (w/v) glucose; the temperature was 28 °C, and the cultivation was performed under continuous illumination at an illumination strength of approximately 25 μE/m^2^ s in a rotary shaker at 120 rpm.^52^ Subsequently, algae were collected through centrifugation at 800*g*, and the concentration was adjusted to OD_750 nm_ = 1. Algae were placed on glass slides in Petri dishes and incubated for 7 days at 28 °C under dim light. Each prepared material sample was placed in a plastic Petri dish that was filled with 60 mL of algal suspension with a concentration of approximately 10^7^ cells/mL. Each treatment was performed three times. After 7 days, the algal suspension was removed from the plastic Petri dish, and the samples to which algae had adhered were photographed. The samples were washed using a water jet containing 20 mL of algal culture medium to remove unattached algal cells. The samples were then immediately immersed in 50 mL of deionized water and subjected to ultrasonic vibration for 5 min to detach the algae attached to the substrate. Subsequently, the deionized water with detached algae was examined using a measuring instrument with high sensitivity to chlorophyll fluorescence—the bbe PhycoProbe. The number of alga cells that had attached themselves to the substrate was determined. The total number of alga cells in each suspension was determined using the following equation

Total algae (number of algae, per mL cm^2^) = average number of alga cells counted using the bbe PhycoProbe (count/mL) × dilution volume (50 mL)/unit area (6.25 cm^2^).

### Characterization

The surface morphologies of the samples were examined using a confocal laser scanning microscope (LEXT OLS5100, Olympus), a scanning electron microscope (ZEISS ULTRA plus), and an atomic force microscope. Bright-field TEM images with mass-thickness contrast were obtained using a JEOL JEM-2010 LaB_6_ transmission electron microscope (at an accelerating voltage of 120 kV). SAXS was conducted using the Bruker NanoSTAR tool, which was equipped with a 2D position-sensitive proportional counter (PSPC) detector (camera length of 1055 nm) and operated at 50 kV per 100 mA. For the AFM observations, a tapping-mode scanning probe microscope was used to obtain images of the thin films and buckled surfaces. These examinations were conducted at room temperature by using an Olympus AC200TS microcantilever attached to a Dimension-3100 AFM device. The setup of the cantilever involved a scan speed of 1 Hz, and the frequency of the tip ranged from 70 to 80 Hz. For the SEM observations, a platinum film was vacuum-sputtered on a sample for 2 min by using a sputter current of 2 mA; this created a film thinner than 2 nm and increased the electrical conductivity of the surface. Images were obtained at an accelerating voltage of 3 kV. The O_2_-RIE treatment for oxidation was performed for 45 s at a radio frequency power of 100 W and a pressure of 75 mTorr. Thermal analysis was conducted using a TA Instrument (DSC-2010). The DSC samples were detected at 20–160 °C under a heating rate of 10 °C/min and nitrogen flow rate of 50 mL/min. Static contact angles were determined using the Phoenix-MT (Surface Electro Optic Co., Ltd., Korea) in air at ambient temperature; 3 μL water droplets were employed. WCA, SA, and CAH were measured using Phoenix MT (A) (Surface Electro Optics, SEO, Korea) at ambient temperature. The volumes of droplets were calculated using the software Surfaceware. The volume of each static CA and SA measurements droplet was 3 and 10 μL, respectively. The value reported was the average of five measurements of the same sample. For measuring SAs, the samples on the stage were tilted from their horizontal position with a tilting velocity of 1 deg/s, until the droplets were influenced by gravity and started to slide, then the SAs were recorded. Advancing and receding contact angles (ACA and RCA) are determined by the drop expansion/contraction method. The ACA and RCA are measured when the sessile droplet is expanding or contracting between 10 and 20 μL by continuously adding or withdrawing a constant volume of a liquid (0.5 μL/s). The difference between the measured values of ACA and RCA is the CAH. To evaluate icephobicity, the samples were subjected to a series of tests. First, a sample was immersed in liquid nitrogen for 1 min to simulate freezing conditions. Subsequently, the sample was sprayed with a generous amount of water mist at room temperature, which caused frost and ice to condense on the surface of the sample. Finally, the frozen samples were heated from room temperature to 100 °C for 3 min in a deicing process. In the experiments demonstrating self-cleaning of liquid and dust, the test samples were tilted by up to 15°; all samples were used only once. Soil particles were employed as submicrometer-sized dust particles. Approximately one to three drops of the test liquid were dropped onto the samples; each drop was approximately 5 μL in volume. Dust particles were placed on the surfaces and then washed off using 5 mL water drops.

## Supporting Information Available

Table of SAXS data (*q**), calculated repeating distance (*L*_0_), film thickness, and thickness-to-*L*_0_ ratio for PS-*b*-PDMS samples (Table S1); schematic of fabrication of buckled pattern on elastomer substrate through dynamic-interfacial-release approach (Figure S1); DSC-detected changes in temperature and heat flow during thermal transitions of PS blocks in the PS-*b*-PDMS diBCPs (Figure S2); XPS signals of the PS-*b*-PDMS films after O_2_-RIE and before and after thermal treatment (Figure S3); AFM height image of the buckled PS-*b*-PDMS samples after removal of the PDMS wetting layer (Figure S4); the laser-scanning confocal microscopy images of the buckled surfaces after biofoulers deposition (Figure S5); TGA result and AFM images of PDMS–Cl brushes grafted on PDMS elastomer substrates (Figure S6); illustration of fabrication of wrinkled SLIPS and its morphologies (Figure S7); CAH and SA of water droplets on various buckled surfaces (Figure S8); the results of chemical and physical resistance tests of buckled PSDS-3039 sample (Figure S9); comparison of green algae settlement on buckled substrates (Figure S10) (PDF)
Movie S1 shows three PS-*b*-PDMS diBCPs buckled samples fabricated using hydrolysis-driven dynamic-release-induced process (MP4)
Movie S2 shows the anti-icing properties and the self-cleaning abilities of the w-SLIPS (MP4)
