# Supplementary material for: Fabrication of Stable Liquid-like Wetting Buckled Surfaces as Bioinspired Antibiofouling Coatings by Using Silicon-Containing Block Copolymers
Source: ACS Appl Mater Interfaces. 2024 Jul 4;16(28):37212–25. doi: 10.1021/acsami.4c06172 (PMC11261564; doi:10.1021/acsami.4c06172)
Supplement: Supplementary file 1 — am4c06172_si_001.pdf [file am4c06172_si_001.pdf]

Supporting Information

Fabrication of Stable Liquid-Like Wetting Buckled Surfaces as Bioinspired Antibiofouling Coatings by Using Silicon-Containing Block Copolymers

*Ting-Lun Chen[1], Ching-Yu Huang[1], Yi-Shan Lai[1], Yi-Chen Chen[1], Yi-Ju Yang[3], Wei-Lung Wang[4], and Han-Yu Hsueh[1,2]**

[1]Department of Materials Science and Engineering, National Chung Hsing University, Taichung, 40227, Taiwan, Republic of China.

[2]Innovation and Development Center of Sustainable Agriculture, National Chung Hsing University, Taichung 40227, Taiwan, Republic of China.

[3]Department of Natural Resources and Environmental Studies, National Dong Hwa University, Hualien 974301, Taiwan, Republic of China.

[4]Department of Biology, National Changhua University of Education, Changhua, 50007, Taiwan, Republic of China.

*To whom correspondence should be addressed.

Tel: 886-4-22840500 ext 506; Fax: 886-4-22857017; E-mail: hyhsueh@nchu.edu.tw

Department of Material Science and Engineering, National Chung Hsing University, Taichung 40227, Taiwan, Republic of China.

Table S1. SAXS data (q^*), calculated repeating distance (L_0), film thickness, and thickness-to-L_0 ratio for PS-*b*-PDMS Samples.

Sample	q^* (nm^{-1})	L_0 (nm)	film thickness (nm)	thickness/L_0
PSDS-4709	0.15326	41.0	430.2	10.5
PSDS-3039	0.16624	37.8	312.5	8.3
PSDS-2885	0.13322	47.2	343.3	7.3

We used SEM cross-sectional observation to confirm the thickness of the PS-*b*-PDMS samples, and their thicknesses were approximately 300 to 400 nm. Furthermore, we utilized SAXS to calculate the repeating distance (L_0) for each PS-*b*-PDMS sample. The repeating distance L_0 was determined from the first peak q^* using the relationship:

$$L_0 = 2\pi/q^*$$

The repeating distance L_0 calculated from SAXS was in the range of 37 to 48 nm for the PS-*b*-PDMS samples. Consequently, the thin film thickness measured was around 7 to 11 times larger than the repeating distance L_0.

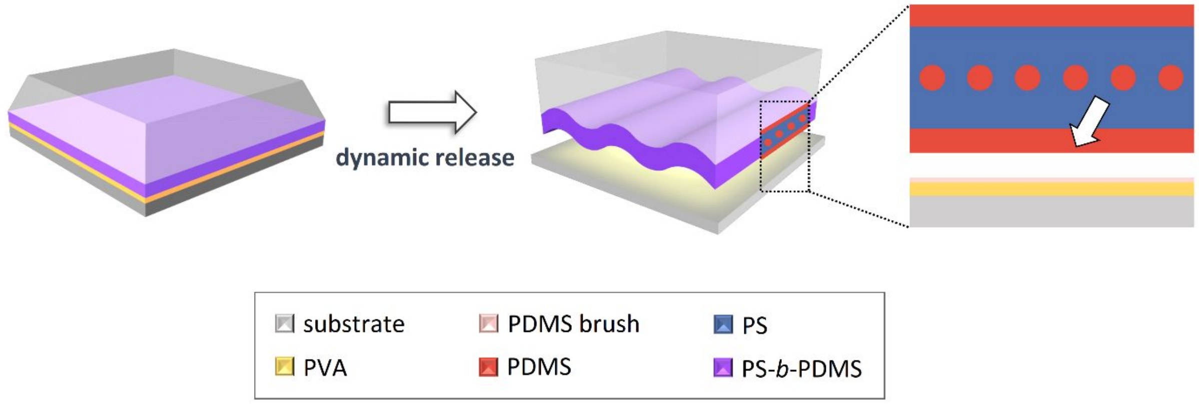

Figure S1. Schematic of fabrication of buckled pattern on elastomer substrate through dynamic-interfacial-release approach. Buckled surface is interface that was originally in contact with PDMS brushes.

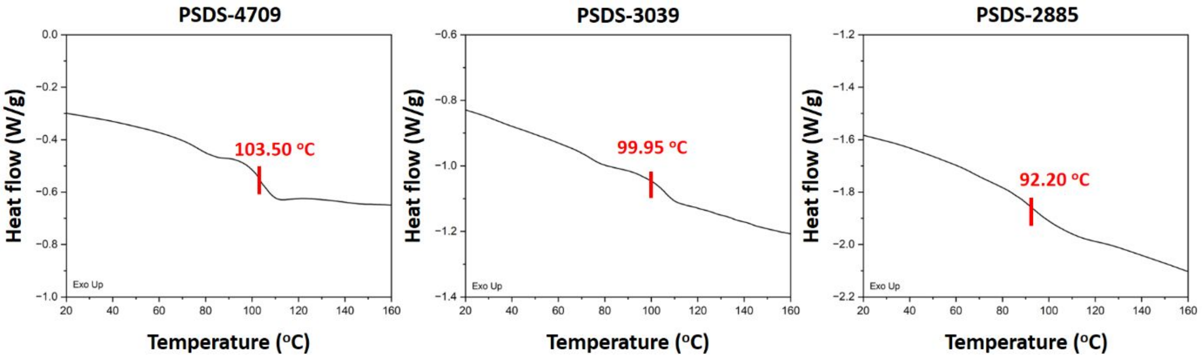

Figure S2. DSC-detected changes in temperature and heat flow during thermal transitions of PS blocks in three PS-*b*-PDMS diBCPs (PSDS-4709, PSDS-3039, and PSDS-2885). PS T_g of diBCP samples was positively correlated with PS molecular weight. T_g ranged between 90 and 105 °C.

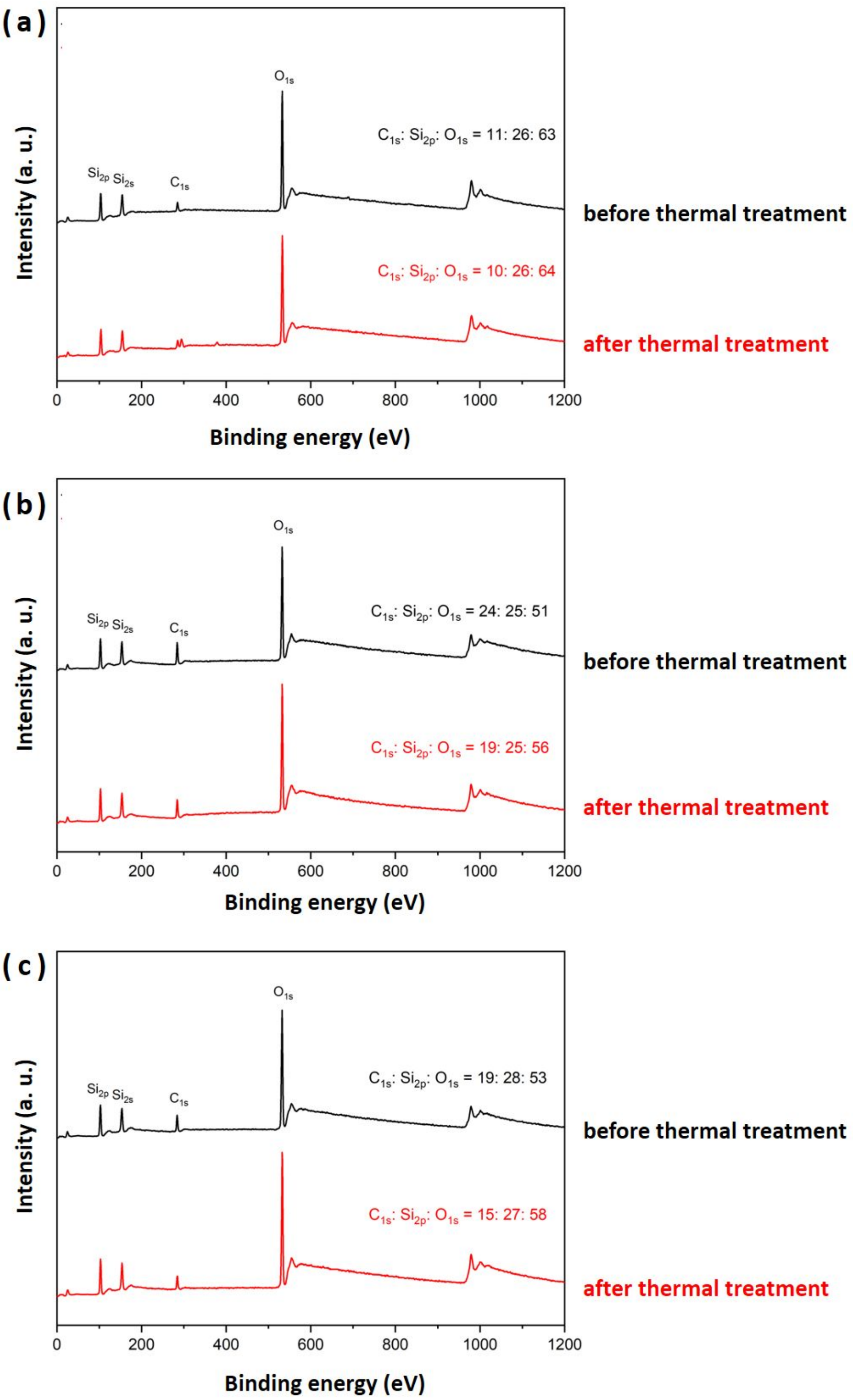

Figure S3. XPS signals of three PS-*b*-PDMS films after O_2-RIE and before and after thermal treatment: (a) PSDS-4709, (b) PSDS-3039, and (c) PSDS-2885. Black and red lines indicate before and after thermal treatment, respectively. Si_{2p}, Si_{2p}, C_{1s}, and O_{1s} peak signals were detected at 102, 153, 285, and 530 eV, respectively.

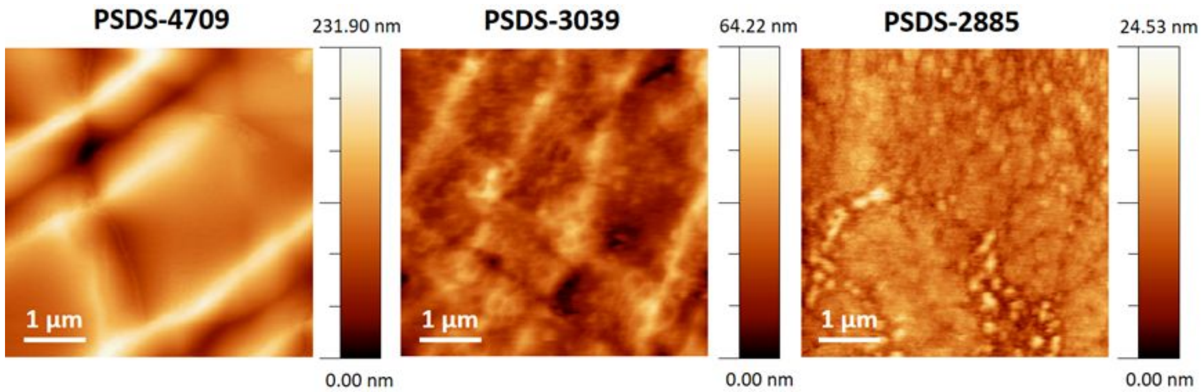

Figure S4. AFM height image of PSDS-4709, PSDS-3039, and PSDS-2885 after removal of the PDMS wetting layer.

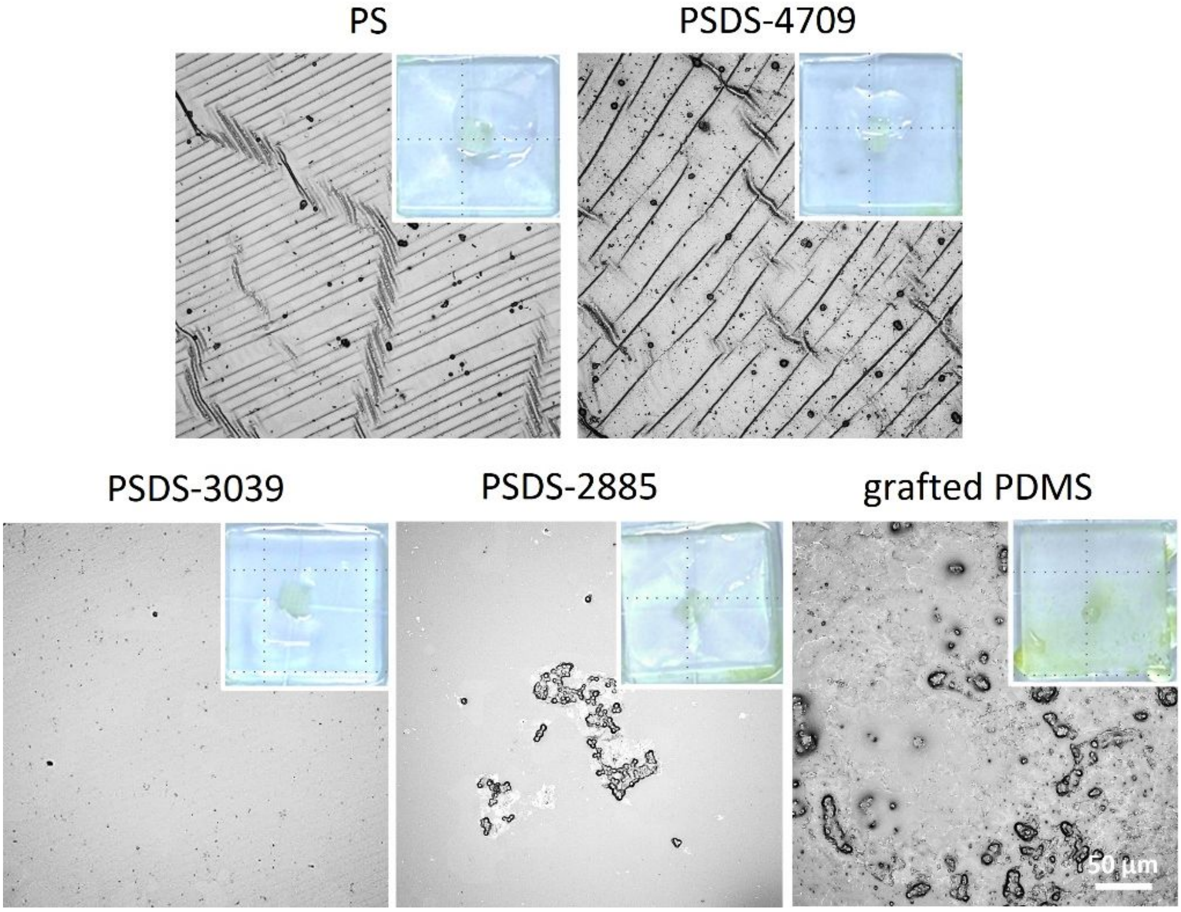

Figure S5. The laser-scanning confocal microscopy images of the buckled PS, PSDS-4709, PSDS-3039, PSDS-2885, and grafted PDMS surfaces after biofoulers deposition. The insets show the corresponding photographs.

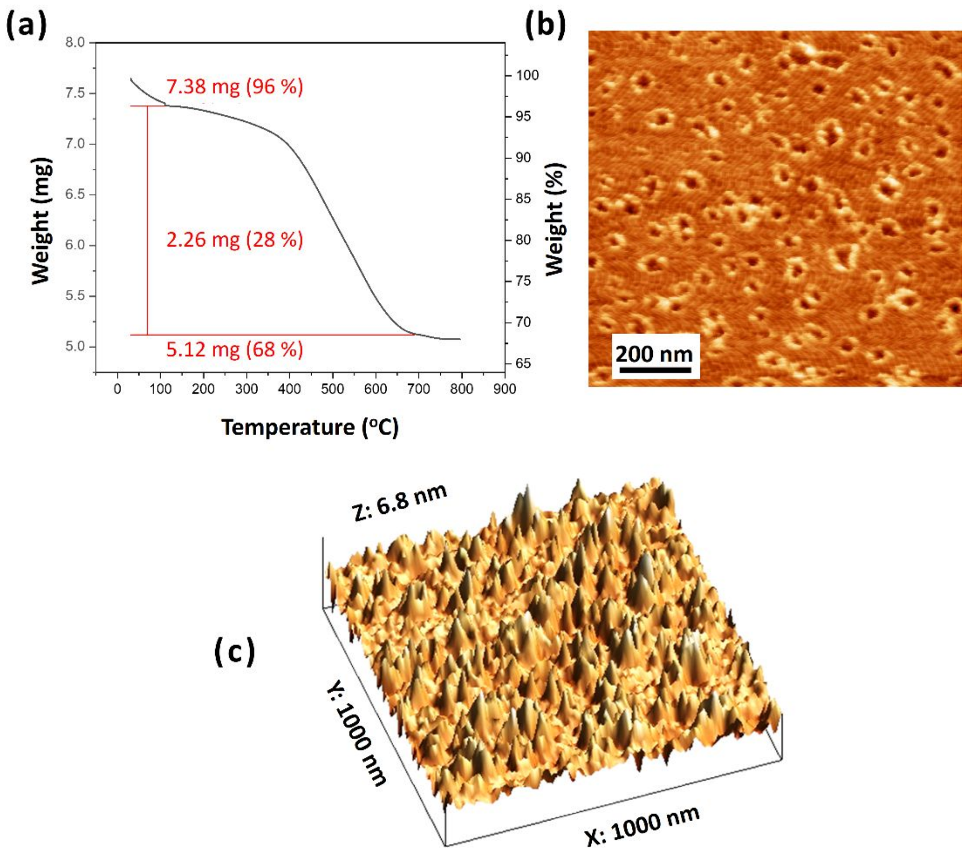

Figure S6. (a) Thermogravimetric Analysis (TGA) curve of the PDMS-grafted substrate. Grafted substrates were heated from ambient temperature to 800 °C at a rate of 10 °C/min under a nitrogen atmosphere. The weight loss due to the decomposition of the grafted PDMS was observed in the grafted substrate but not in the ungrafted sample. The grafting density (σ) was calculated using the equation $\sigma = \Delta W \times N_A/(A \times Mn)$, where ΔW is the difference in weight loss after the PDMS brushes decomposing, N_A is Avogadro constant with an exact value of 6.022×10^{23} mol^{-1}, A is the surface area of the substrate, and Mn is the number-average molecular weight of PDMS-Cl. As a result, the grafting density of PDMS brushes on the substrate was determined to be 1.81×10^{18} molecules/cm^2. (b) AFM height image of PDMS–Cl brushes grafted on PDMS elastomer substrates. Scan area is 1×1 μm^2. (c) 3D AFM image of the corresponding PDMS-grafted substrate. The PDMS brushes were composed of particle islands, and their average roughness was around 0.525 nm. Clearly, the grafting sites were spaced widely and thus not conducive to a high grafting density.

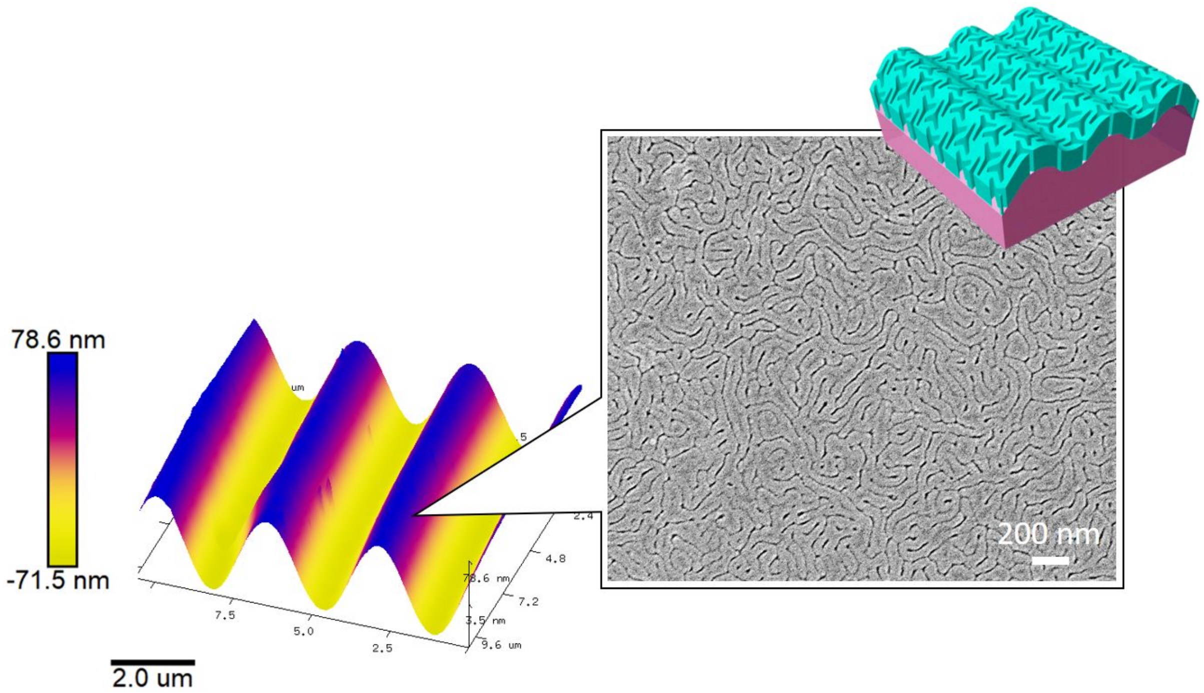

Figure S7. 3D AFM image of repeated and oriented microwrinkled patterns (left). SEM micrograph of microwrinkled nanoporous PS with co-continuous nanochannels on the surface before infiltration of silicone oil.

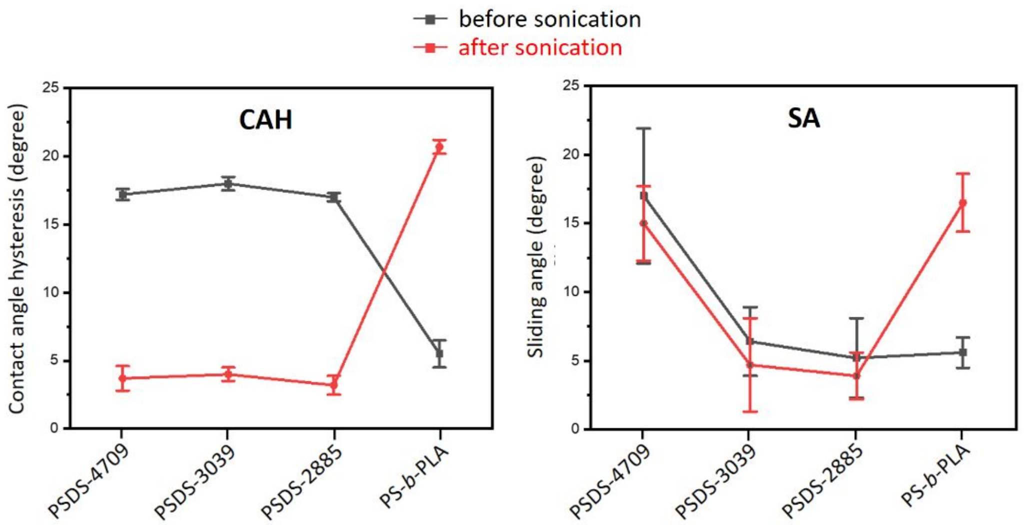

Sample	PSDS-4709		PSDS-3039		PSDS-2885		PS-*b*-PLA	
Unit: degree	before	after	before	after	before	after	before	after
WCA	100.0 ± 0.9	101.2 ± 4.2	102.3 ± 2.5	103.4 ± 3.1	107.5 ± 3.6	105.3 ± 1.4	119.8 ± 3.5	100.0 ± 4.0
ACA	106.3 ± 0.4	105.6 ± 1.8	111.4 ± 0.9	106.3 ± 2.5	112.3 ± 0.4	108.4 ± 2.7	108.2 ± 0.4	108.5 ± 8.5
RCA	89.1 ± 0.0	101.9 ± 1.5	93.4 ± 0.6	102.4 ± 2.2	95.2 ± 0.1	105.1 ± 2.8	102.6 ± 0.7	87.8 ± 2.0
CAH	17.2 ± 0.4	3.7 ± 0.9	18.0 ± 0.5	4.0 ± 0.5	17.0 ± 0.3	3.2 ± 0.7	5.5 ± 1.0	20.7 ± 0.5
SA	17.0 ± 4.9	15.0 ± 2.7	6.4 ± 2.5	4.7 ± 3.4	5.2 ± 2.9	3.9 ± 1.7	5.6 ± 1.1	16.5 ± 2.1

Figure S8. Contact angle hysteresis (CAH) and sliding angle (SA) of water droplets on various slippery buckled surfaces, including buckled PSDS-4709, PSDS-3039, PSDS-2885, and buckled PS-*b*-PLA before and after ultrasonic vibration. The table details water contact angle (WCA), advancing contact angle (ACA), receding contact angle (RCA), CAH, and SA. Each data point represents the average of five tests.

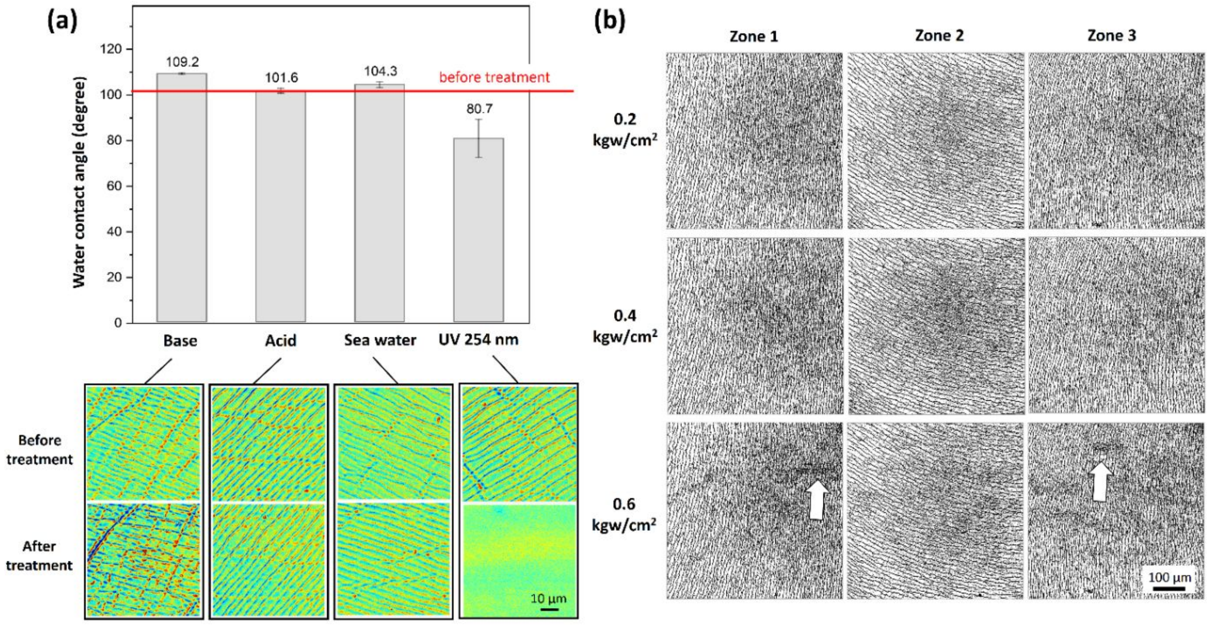

Figure S9. (a) Water CAs of buckled PSDS-3039 after chemical immersion in different solvents and UV light exposure. The bottom section illustrates the corresponding surface morphologies before and after treatment, as observed using laser-scanning confocal microscopy. (b) Images obtained by laser-scanning confocal microscopy of buckled PSDS-3039 under compressive stresses ranging from 0.2 to 0.6 kgW/cm².

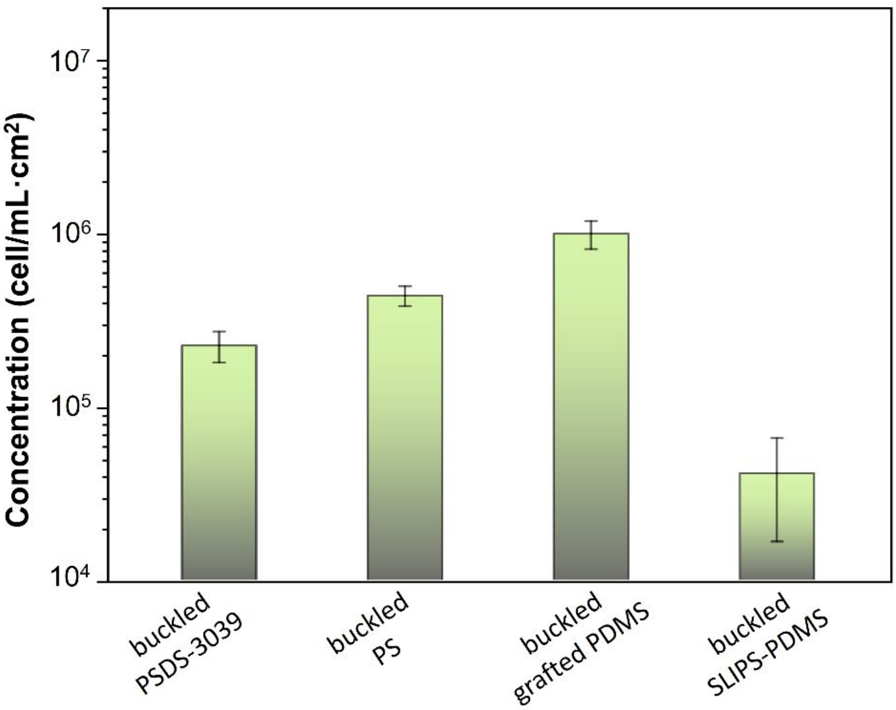

Figure S10. Comparison of green algae settlement on buckled substrates, including PSDS-3039, PS, PDMS-grafted PDMS, and gyroid surface infused with silicone oil (i.e., SLIPS-PDMS).
